# Red-light modulated *ortho*-chloro azobenzene photoswitch for peptide stapling *via* aromatic substitution†

**DOI:** 10.1039/d3cb00176h

**Published:** 2023-10-19

**Authors:** Mia Kapun, F. Javier Pérez-Areales, Nicola Ashman, Pamela J. E. Rowling, Tim Schober, Elaine Fowler, Laura S. Itzhaki, David R. Spring

**Affiliations:** a Yusuf Hamied Department of Chemistry, University of Cambridge Cambridge CB2 1EW UK spring@ch.cam.ac.uk; b Department of Pharmacology, University of Cambridge Tennis Court Road CB2 1PD Cambridge UK

## Abstract

The application of peptide stapling using photoswitchable linkers has gained notable interest for potential therapeutic applications. However, many existing methodologies of photoswitching still rely on the use of tissue-damaging and weakly skin-penetrating UV light. Herein, we describe the development of a tetra-*ortho*-chloro azobenzene linker that was successfully used for cysteine-selective peptide stapling *via* S_N_Ar. This linker facilitates precise photocontrol of peptide structure *via trans* to *cis* isomerisation under red light irradiation. As a proof-of-concept, we applied the developed peptide stapling platform to a modified PMI peptide, targeting the inhibition of MDM2/p53 protein–protein interaction (PPI). Biophysical characterisation of the photoswitchable peptide by competitive fluorescence polarisation showed a significant difference in affinity between the *trans* and *cis* isomer for the p53-interacting domain of the human MDM2. Remarkably, the *cis* isomer displayed a >240-fold higher potency. To the best of our knowledge, this is the highest reported difference in binding affinity between isoforms of a photoswitchable therapeutic peptide. Overall, our findings demonstrate the potential of this novel photoswitchable peptide stapling system for tuneable, selective modulation of PPIs *via* visible-light isomerisation with deeply-tissue penetrating red light.

## Abstract 2

Development and proof-of-concept for a photoswitchable peptide staple with the ability to trigger a major change in binding affinity upon visible light irradiation.

## Graphical abstract

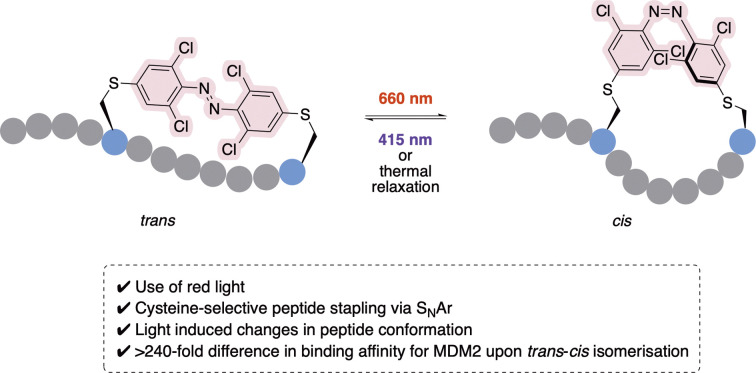

## Introduction

Photopharmacology is an emerging therapeutic area which utilises light to alter the molecular structure of a drug. Such reversible or irreversible light-promoted changes can provide a rapid and non-invasive spatiotemporal control of drug activity. Hence, light can be used to activate a photoswitchable drug at its site of action to reduce off-target toxicity and improve the therapeutic index.^1,2^ Thus, light-activated approaches can overcome safety-related failures that commonly lead to high attrition rates of drugs in clinical development, limiting the approval of new medicines.^3,4^

Numerous molecular photoswitches have been previously explored, including azobenzene (AB) derivatives. ABs are easily synthesised and readily interchanged between their thermally stable *trans* and metastable *cis* forms.^5^ Due to their favourable photophysical properties, AB photoswitches have been applied to modulate a variety of biological systems,^5^ including peptides,^6^ proteins,^7^ lipids,^8^ oligonucleotides,^9^ and carbohydrates.^10^

**Fig. 1:**
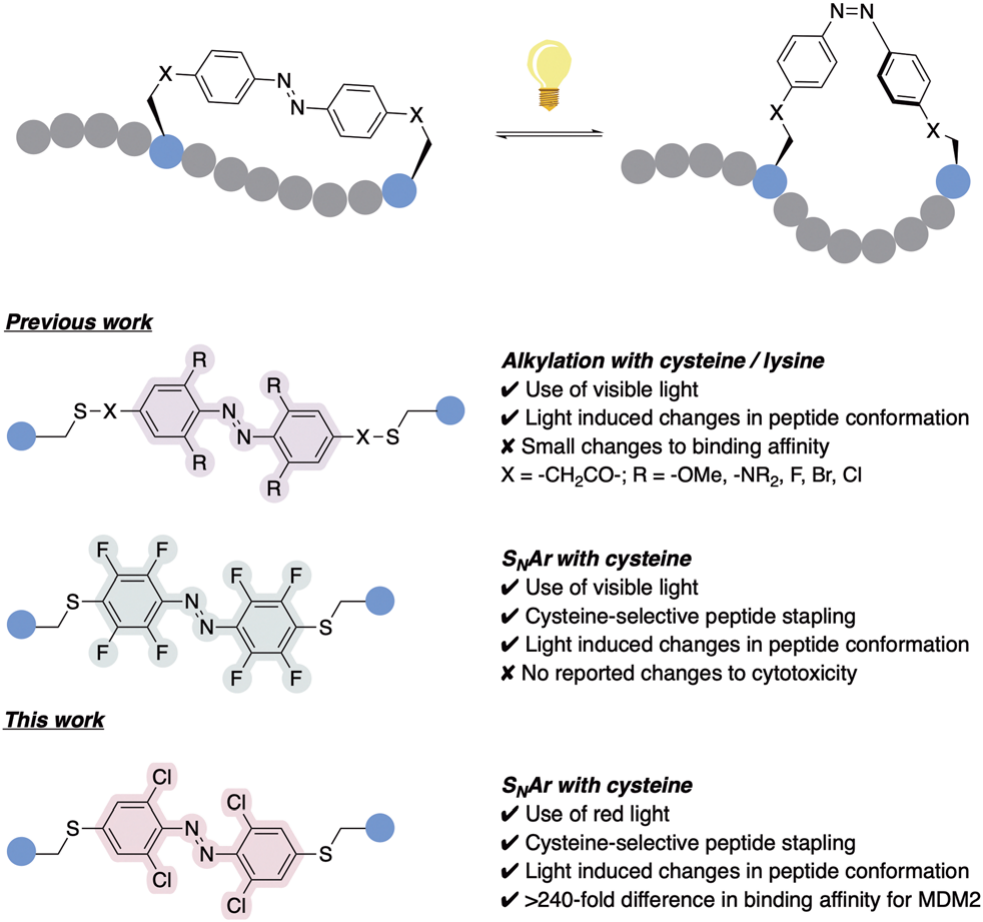
Previous work utilising alkylation and S_N_Ar for the incorporation of *ortho*-substituted ABs for peptide stapling,^14–19^ together with our *ortho*-chloro AB red-light activated peptide stapling *via* cysteine-S_N_Ar methodology.

The application of photoswitches to peptide stapling has previously been studied, demonstrating a precise control of peptide structure and function.^6,7,11^ However, despite the recent advances, most AB systems utilised for stapling require the use of skin-damaging and weakly tissue-penetrating UV light, limiting its utility.^12^ Efforts towards ‘next-generation’ AB-incorporated peptides have shown the use of visible-light photoswitching as a promising strategy for the development of peptides with therapeutic potential, specifically using red light which deeply penetrates the human tissue in comparison to other visible light wavelengths.^13^ This was achieved through the integration of AB systems with tuned photochemical properties, which for example emerged from the introduction of *ortho* substituents to the azobenzene rings, such as methoxy groups,^14^ amines,^15^ fluorines,^16,17^ bromines or chlorines (Fig. 1).^18^ However, most of the reported examples rely on alkylation or backbone incorporation, with the exception of one fluorine-substituted example,^19^ or the utilisation of S_N_Ar for protein modification.^20^ Whilst such ABs afforded significant changes in peptide conformation upon isomerisation,^18,21^ the effect of this conformational change on binding affinity remained limited, with many cases showing limited ability to switch the biological activities between the isomeric peptide forms.^17,19^ Conversely, AB systems have been successfully applied to modulate antibody–antigen interaction for protein purification.^22^ Hence, there is a requirement for the development of effective, tuneable photoswitchable peptide staples which provide sufficient on–off switching of biological properties triggered by a conformational change.

Transcription factor p53 is a tumour suppressor, and its low cellular levels are often associated with cancer development. As an E3 ubiquitin ligase, MDM2 downregulates p53, repressing the p53 regulatory control on transcriptional activity, which ultimately leads to tumour growth.^23^ Inhibition of the p53/MDM2 interaction has been shown to efficiently rescue p53 from degradation, thus recovering its tumour suppressor activity. In fact, there are ongoing clinical trials of peptide inhibitors targeting the PPI between p53 and MDM2. Notably, ALRN-6924 was the first stapled peptide against the p53/MDM2 interaction to enter the clinic, however later studies revealed a sub-optimal toxicity profile.^24–26^ Thus, there is an unmet need for the discovery of novel stapled peptides which inhibit the p53-MDM2 interaction with improved safety profile.

Herein, we describe the development of a novel red-light photoswitchable platform for peptide stapling based on Cysteine-S_N_Ar, which was successfully applied towards the inhibition of the p53/MDM2 interaction. To this end, we initially screened three different ABs for peptide stapling, which were characterised for their reactivity and photochemical properties. The selected tetra-*ortho*-chloro azobenzene 3 was then used for peptide stapling of PMI, a well-known PPI inhibitor of p53/MDM2, as a proof-of-concept for the ability of the staple platform to efficiently provide an on–off switch under highly-penetrating visible-light irradiation.

## Results and discussion

### Design and synthesis of azobenzene staples

Photocontrol of peptides is mostly based on changes in geometry and an interplay between flexibility and rigidity. S_N_Ar displacement of fluorine with cysteine affords rigid linkers with the potential to conduct significant geometrical changes onto the peptide, thereby yielding to major changes in biological activity between its isomers.^19,20^ We envisioned three different fluorinated azobenzene candidates for peptide stapling. The selection of these candidates was made on the basis of their photochemical properties, given that they undergo *trans* to *cis* isomerisation upon visible-light irradiation, either with green light for ABs 1 and 2 or red light for AB 3.^19,27–30^ Azobenzene staples (1–3) were synthesised in a single step, following the procedure developed by John *et al.*,^31^ from the corresponding anilines (Table 1). The products were isolated as a mixture of *cis* and *trans* isomers upon purification.

**Table 1:** Synthesis of azobenzene linkers 1, 2 and 3

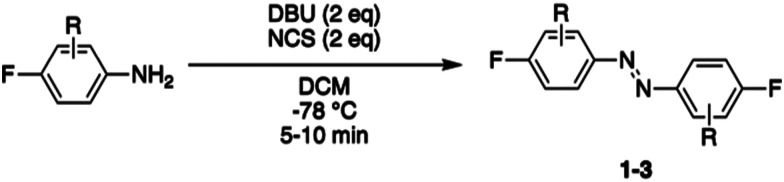		
Entry	Aniline	Yield (%)
1	2,3,4,5,6-F (1)	38
2	2,4,6-F (2)	42
3	4-F-2,6-Cl (3)	24

### Reactivity assessment

Synthesis was followed by cysteine reactivity assessment to explore their potential for undergoing the desired S_N_Ar while avoiding over reactivity. Hence, staples 1–3 were incubated with *N*-acetyl cysteine (2 eq.), Tris base (100 eq.) and tris(2-carboxyethyl)phosphine hydrochloride (TCEP·HCl) (10 eq.) at room temperature in DMF, to mimic peptide stapling conditions (see Table S1, ESI† for detail). The products formed were analysed by LCMS, ^1^H and ^19^F NMR. Staple 1 afforded the desired disubstituted product, but despite literature precedence suggesting that fluorinated azobenzene 1 would exclusively react in the *para* position,^19^ unwanted reactivity at the *ortho* position was observed. Indeed, the formation of tetra- and penta-substituted side-products was further confirmed upon addition of cysteine in excess. Likewise for peptide staple 2, a mixture of mono-, di- and tri-substituted products was observed by LCMS and NMR analysis. Cysteine conjugation with staple 3 was deemed the most promising since reactivity was exclusively observed in the *para* position.

To probe cysteine selectivity and compatibility with canonical amino acids, staples 1–3 were incubated with *N*-acetyl lysine, under similar conditions (see Table S1, ESI† for detail). Conversely to previous reports for the decafluorinated biphenyl system,^32^ decafluorinated 1 and hexafluorinated 2 reacted with lysine, affording a range of undesired lysine mono- and di-substituted products. Hence, these staples were considered unsuitable for their application of cysteine peptide stapling in the presence of lysine residues. However, staple 3 exhibited a residual reactivity with lysine, thus showing a clear preference for cysteine residues.

The exclusive *para*-reactivity and high cysteine selectivity made peptide staple 3 an ideal candidate for generating photoswitchable stapled peptides through direct cysteine arylation, allowing an efficient cysteine conjugation.

### Photochemical characterisation of cysteine-conjugated *ortho*-chloro azobenzene staple

**Fig. 2:**
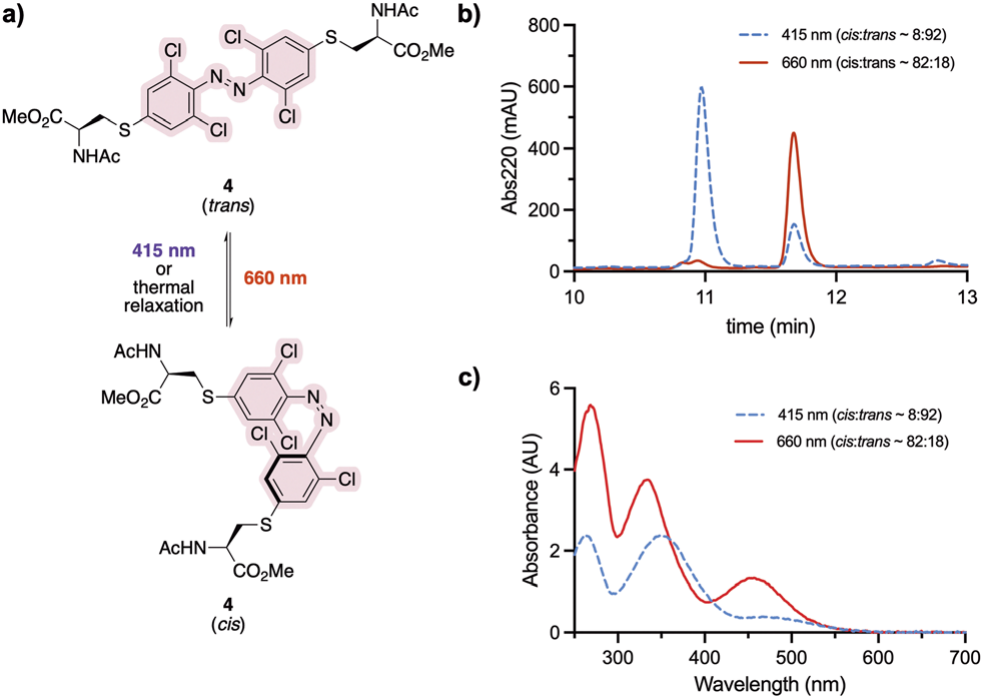
(a) Photoisomerisation studies of cysteine-conjugated staple 4. (b) *trans*–*ci*s isomerisation of 4 (500 μM in H_2_O/MeCN 50 : 50) followed by analytical HPLC. (c) Isomer UV-vis analysis of 4 (500 μM in H_2_O/MeCN 50 : 50). The spectra were recorded upon 30 min and 90 min irradiation with 415 nm and 660 nm LED lights, respectively.

Staple 3 was reacted with *N*-acetyl cysteine to form the model system 4, mimicking a stapled peptide, for further investigation of its isomerisation properties (Fig. 2a). Visible light isomerisation of 4 was carried out with purple and red light, and the *cis*/*trans* isomer ratio determined by HPLC and spectrophotometry analysis. *trans* to *cis* isomerisation of 4 was successfully achieved upon 90 minute irradiation with red light (*λ* = 660 nm, 20.88 μW mm^−2^), yielding 82% of the *cis* isomer, while *cis* to *trans* isomerisation upon 30 minute irradiation purple light (*λ* = 415 nm, 15.60 μW mm^−2^) led to 92% conversion to the *trans* isomer (Fig. 2b and c).

### Design and synthesis of stapled peptide

**Fig. 3:**
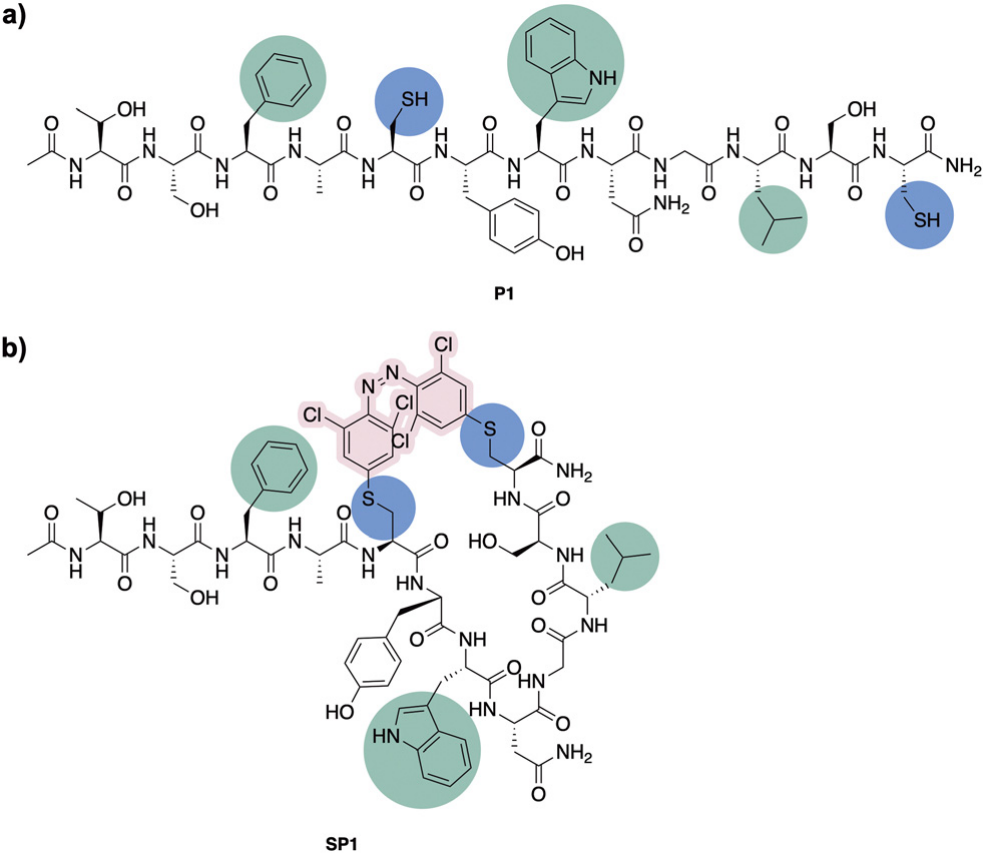
(a) Peptide stapling precursor P1. Highlighted in blue the incorporated Cys residues and in green the amino acid residues involved in the interaction with MDM2. (b) Structure of the (*i*, *i* + 7) azobenzene-containing stapled peptide SP1.

Having confirmed the desired isomerisation of model system 4 under visible light, staple 3 was incorporated into a derivative of a potent p53/MDM2 inhibitor (PMI; TSFAEYWNNLSP).^33^ Consistent with our experience in stapling, cysteine substitutions were made at positions 5 and 12 to facilitate peptide stapling at (*i*, *i* + 7) positions (Fig. 3).^34,35^ Previous reports suggest replacement of these residues allow for the incorporation of the staple without disrupting the interaction with MDM2, mainly driven by the “hot spot” residues, namely Phe3, Trp7 and Leu10.^33^

*N*-Ac-capped PMI with a C-terminal amide, referred to from this point as PMI, as well as the PMI-derived precursor for peptide stapling P1, were manually synthesised by Fmoc-SPPS on a low-loading Rink Amide MBHA resin (see ESI† for more detail). The crude peptides were purified by preparative reverse phase HPLC. P1 was subsequently stapled *via* S_N_Ar cysteine conjugation with azobenzene staple 3 to yield the stapled peptide SP1 in 38% yield upon HPLC purification.^32^ The resulting stapled peptide was characterised by LCMS and analytical HPLC, with the two isomers displaying distinct retention times. Peptide stapling with ABs 1 and 2 was also attempted under the same reaction conditions, for the sake of comparing the effect of having different substituents on the AB of the stapled peptide. Unsurprisingly, reactivity with multiple peptide strands yielded complex mixtures and the desired stapled peptides were unsuccessfully isolated.

### Photochemical characterisation of SP1

**Fig. 4:**
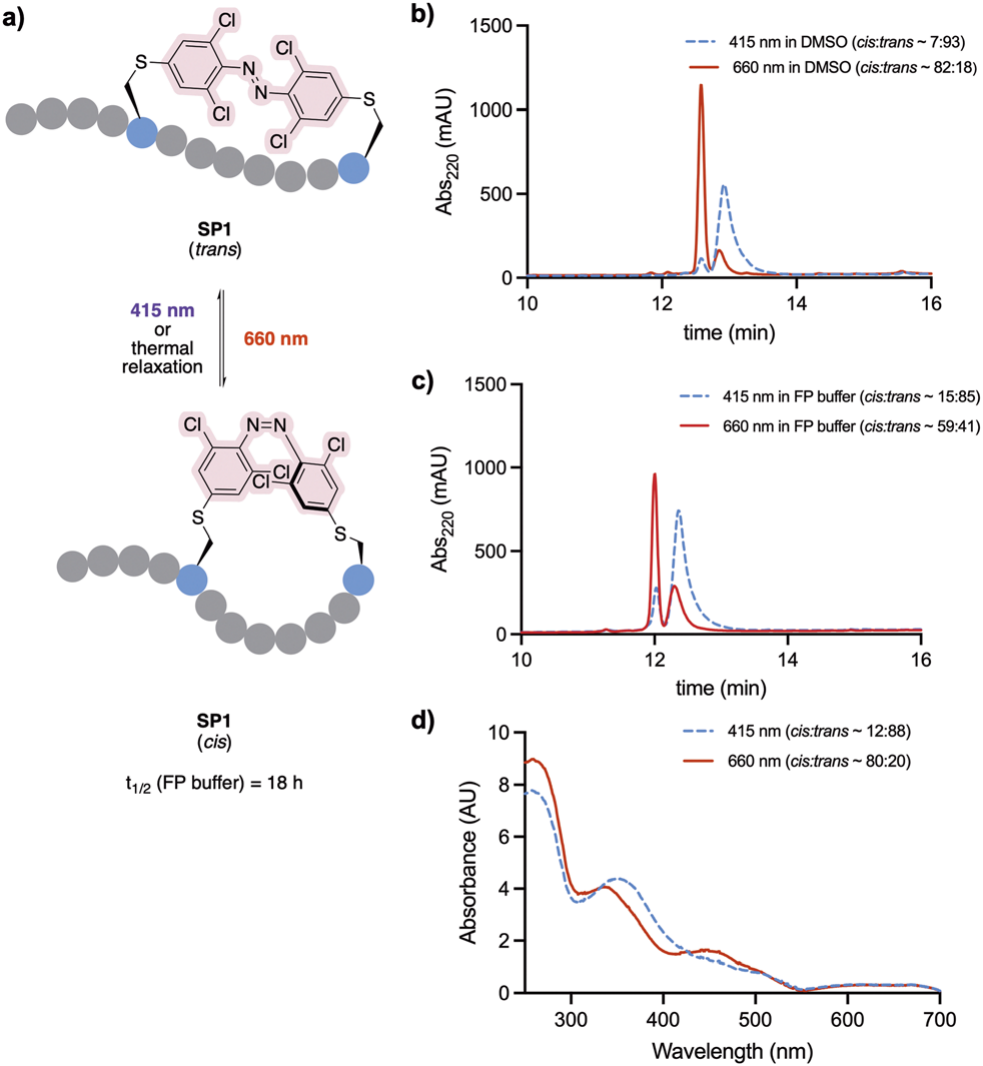
(a) Photoisomerisation of stapled peptide SP1. (b) *trans*–*cis* isomerisation of SP1 in DMSO followed by analytical HPLC. (c) *trans*–*cis* isomerisation of SP1 in and FP buffer followed by analytical HPLC. (d) UV-vis spectrum of SP1 (500 μM H_2_O/MeCN 50 : 50). All the spectra were recorded upon 30 min and 90 min irradiation with 415 nm and 660 nm LED lights, respectively.

The conformational behaviour of the stapled peptide SP1 was examined (Fig. 4). Upon purification, SP1 was isolated as a mixture of *cis* and *trans* isomers, predominantly existing in its thermally stable *trans* form. Initially, isomerisation studies of the stapled peptide SP1 were carried out in a variety of solvent systems. Successful red-light induced isomerisation towards the formation of the metastable *cis* isomer of SP1 was achieved in DMSO, H_2_O/MeCN 50 : 50 and FP buffer (PBS, 0.05% (v/v) Tween-20, 3% DMSO), in 82%, 80% and 59% yields, respectively (Fig. 4b and c; and ESI†). UV-vis analysis further confirmed distinct absorption spectra of each isomer for SP1 (Fig. 4d).

**Fig. 5:**
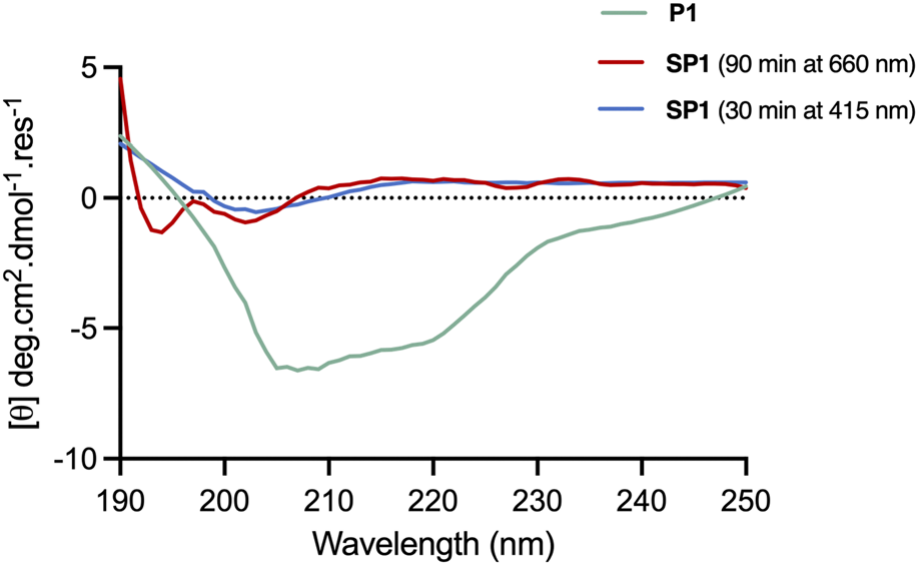
CD spectra of the linear peptide P1 in 50 : 50 H_2_O/MeCN, and stapled peptide SP1 in MeCN, recorded across 190–250 nm. *In situ* isomerisation of SP2 was carried out upon 30 min and 90 min irradiation with 415 nm and 660 nm LED lights, respectively.

The conformational changes induced by red-light irradiation and *trans*–*cis* isomerisation of SP1 peptide were analysed by circular dichroism (CD) spectroscopy and compared to the precursor peptide P1 (Fig. 5). The spectrum of P1 showed some contribution from α-helical structures, with signals around 208 nm and 222 nm, as well as contributions from disordered regions. Both isomers of stapled SP1 displayed a prevalence of disordered regions, with apparent changes to the secondary structure upon *in situ* red-light isomerisation. Indeed, a minimum near 193 nm for the *cis* isomer might presumably reflect the presence of a β-turn region.^36^

The half-life of the metastable *cis* isomer was analysed in FP buffer to estimate its biological applicability. The back-isomerisation of *cis*SP1 was determined to be 18 hours at room temperature (see Fig. S3, ESI† for more detail). As continuous exposure to light to activate the peptide into its active form is challenging *in vivo*, the stability of *cis*SP1 in aqueous buffer is of therapeutic relevance.

### Stability studies

**Fig. 6:**
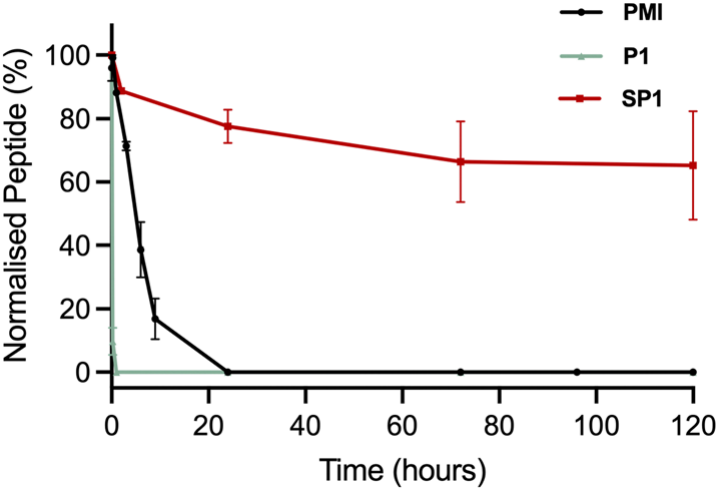
Stability of the precursor peptide P1, the parent PMI and SP1 in human serum at 37 °C, over 5 days, monitored by HPLC at 220 nm.

The stability of SP1 in human serum was investigated and compared to the stability of its linear precursor P1 and the parent PMI (Fig. 6). The stability of the peptides at 37 °C in human serum was monitored throughout 5 days by HPLC. As expected for stapled peptides, SP1 showed markedly improved stability compared P1 and PMI, which can be attributed to the conformational rigidity introduced *via* stapling.

### Biophysical characterisation

**Fig. 7:**
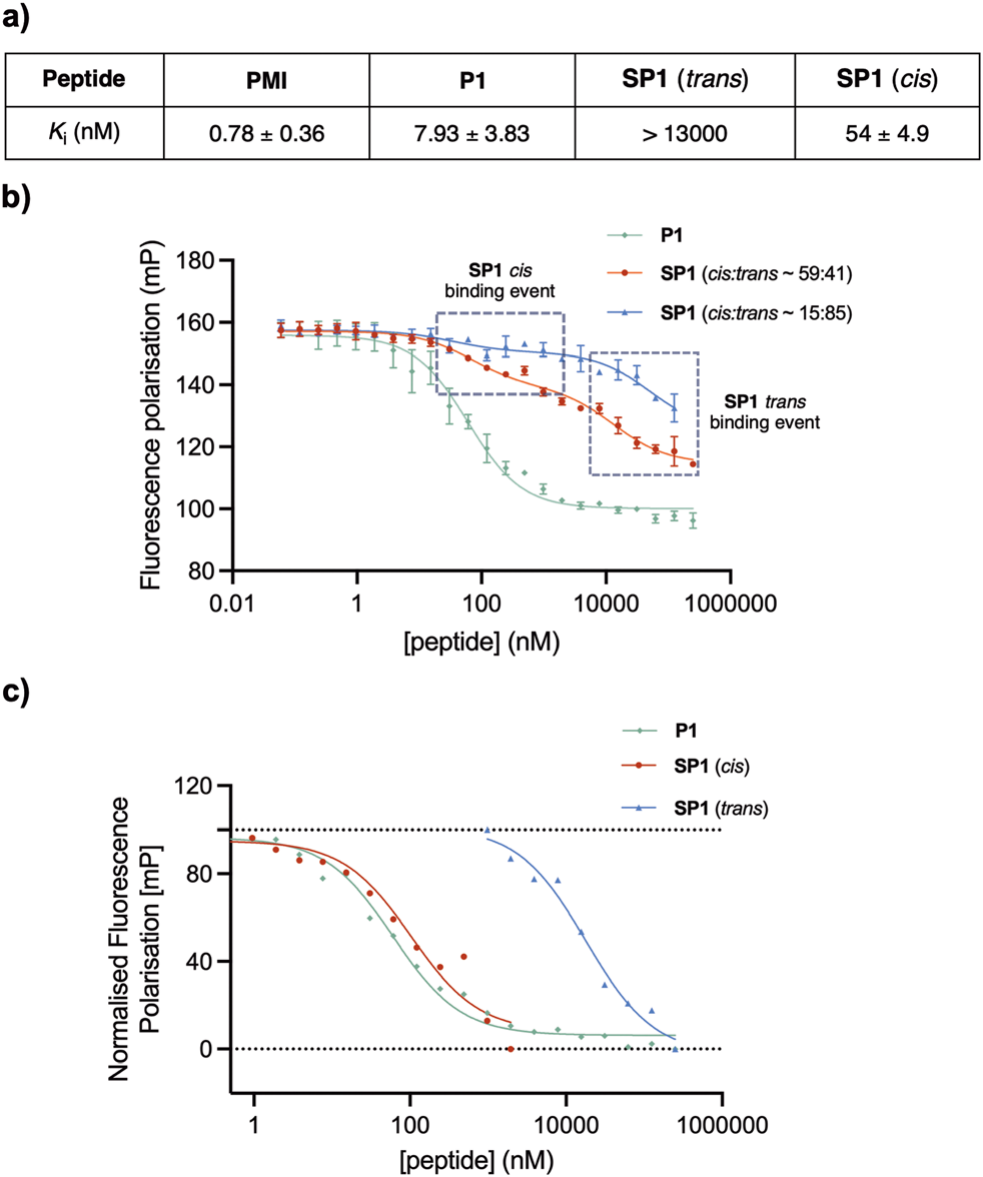
(a) Binding affinities of PMI, P1 and SP1 for MDM2. The *K*_i_ values are reported as the average of two or three biological replicates each performed as a triplicate and the error bars shown represent the standard error of the mean, determined from the fit for each of the independent experiments and subtracted from the average *K*_i_ value. (b) Competitive FP assay of P1 and SP1. Each data point is arithmetic mean of two or three biological replicates each performed as a triplicate and the error bar shown are standard errors of the mean. *In-situ* isomerisation of SP1 was carried out upon 30 min and 90 min irradiation with 415 nm and 660 nm LED lights, respectively. (c) Normalised FP curves of *cis* and *trans*SP1 individual binding events, extracted from the SP1 (*cis* : *trans* ∼ 59 : 41) fitted curve, and the normalised FP of P1 for comparison.

To assess the binding affinity of the precursor peptide P1 and the photoswitchable stapled peptide SP1 for MDM2, a competitive fluorescence polarisation (FP) assay was carried out using the p53-interacting domain of human MDM2 (MDM26–125). Initially the dissociation constant (*K*_d_) of a well-known MDM2 inhibitor TAMRA-labelled peptide (FP tracer peptide; TAMRA-RFMDYWEGL-NH_2_) was determined using a direct FP assay, giving a value of 7.6 nM, which was consistent with the previously described.^34,37^ The inhibitory constants (*K*_i_) for all peptides were subsequently determined. In comparison to PMI, which was structurally optimised for the inhibition of the p53/MDM2 interaction, the linear peptide P1 was found to have a reduced binding affinity upon the introduction of two cysteine residues (Fig. 7a). This can be attributed the loss in α-helicity observed for P1, in comparison to that of PMI,^34^ measured by CD analysis. To induce *in situ* isomerisation, SP1 was irradiated with red or purple light in FP buffer prior to commencing the experiment. Given the limited isomerisation upon red-light irradiation in FP buffer, which was previously determined to yield an isomer ratio of ∼59 : 41 *cis*/*trans*, two different binding events were observed for SP1, with each binding event corresponding to the binding of a single isomer present within the sample (Fig. 7b). The *K*_i_ values for each isomer were determined from their corresponding inflection points in the fitted sigmoidal curve. Pleasingly, the *trans* and *ci*s isomers of SP1 displayed significantly different binding affinities for MDM2, with *K*_i_ switching from >13 000 nM (SP1*trans*) to 54 ± 4.9 nM (SP1*cis*). Thus, highlighting the success of the developed on–off photoswitchable platform, which elicited a remarkable >240-fold increase in affinity upon *trans* to *cis* isomerisation. The two different binding events corresponding to the binding of each of the isomers (∼59 : 41 ratio) were extracted and normalised to facilitate visualisation of the binding affinity in comparison with the reference peptide P1 (Fig. 7c). Despite incomplete conversion to the *cis* isomer in the FP assay buffer, yielding a limited isomer ratio of ∼59 : 41 *cis*/*trans*, we can estimate that this mixture is still ∼143-fold more potent than the *trans* isomer.

## Conclusions

In this work, we developed a red-light photoswitchable platform, which is easily accessible in a single reaction, for peptide stapling *via* displacement of fluorine with cysteine residues and demonstrated its great potential in the photopharmacology field. Firstly, we screened three different azobenzene systems for their ability to photoisomerise with visible light and their cysteine-selective reactivity *via* S_N_Ar. Secondly, based on their practicability for stapling, we selected the tetra-*ortho*-chloro azobenezene 3 for further studies. Linker 3 was successfully incorporated into the PMI derivative P1 which originated from the well-known MDM2/p53 interaction inhibitor PMI, *via* cysteine-selective S_N_Ar to form the stapled peptide SP1. The success of this photoswitchable stapled peptide was evidenced by both photochemical and biophysical characterisations. SP1 was able to *cis* isomerise up to 82%, with such a conformational change eliciting a sufficient shift in peptide structure that ultimately led to an exquisite swich on–off capability of MDM2 binding. *cis*SP1 displayed a >240-fold stronger affinity for MDM2 than its *trans* counterpart. Of note, this remarkable switch in affinity represents the highest described, to our knowledge, for a photoswitchable therapeutic stapled peptide between its different isomers. Altogether, the tetra-*ortho*-chloro azobenzene 3 has demonstrated a great potential as a photoswitch for cysteine-selective peptide stapling that can be explored for further applications in photopharmacology, with the potential to enable outstanding spatiotemporal control of activity as well as improved peptide stability.

## Contributions

M. K. was involved in investigation, visualisation and data analysis. F. J. P. A. was involved in supervision. N. A. was involved in investigation. P. R. was involved in data analysis and supervision. T. S. was involved in supervision. E. F. was involved in conceptualisation. L. I. was involved in supervision. D. R. S. was involved in conceptualisation and supervision. Writing – original draft, M. K.; writing – review and editing, all authors.

## Conflicts of interest

There are no conflicts to declare.

## Supplementary Material

CB-005-D3CB00176H-s001
