# Supplementary material for: Red-light modulated ortho-chloro azobenzene photoswitch for peptide stapling via aromatic substitution
Source: RSC Chem Biol. 2023 Oct 19;5(1):49–54. doi: 10.1039/d3cb00176h (PMC10763561; doi:10.1039/d3cb00176h)
Supplement: CB-005-D3CB00176H-s001 [file CB-005-D3CB00176H-s001.pdf]

Electronic Supplementary Material (ESI) for RSC Chemical Biology.
This journal is © The Royal Society of Chemistry 2023

SUPPORTING INFORMATION

Red-light modulated *ortho*-chloro azobenzene photoswitch for peptide stapling via aromatic substitution

Mia Kapun,[a] F. Javier Pérez-Areales,[a] Nicola Ashman,[a] Pamela J. E. Rowing,[b] Tim Schober,[a] Elaine Fowler,[a] Laura S. Itzhaki [b] and David R. Spring*[a]

Table of Contents

1 Figures S1-S5 and Tables S1-S4 3

2 General Experimental Details 8

2.1 Synthetic Procedures 10

2.2 Cysteine and Lysine Reactivity Assessment 16

3 NMR Spectra 25

4 Peptide synthesis, analysis, and stapling procedures 33

4.1 Peptide Synthesis 33

4.2 Peptide Analysis 34

4.3 LCMS Spectra 35

4.4 HPLC Spectra 36

5 Characterisation of Isomerisation 39

5.1 Light-Induced Isomerisation Studies 39

6 Stability Studies 39

6.1 Half-Life Studies 39

6.2 Serum Stability Studies 39

7 Fluorescence Polarisation (FP) Assay 39

7.1 MDM2 Fluorescence Polarisation Binding Assay 39

7.2 MDM2 Competitive Fluorescence Polarisation Binding Assay 40

8 Circular Dichroism (CD) Spectroscopy 41

9 References 42

1 Figures S1-S5 and Tables S1-S4

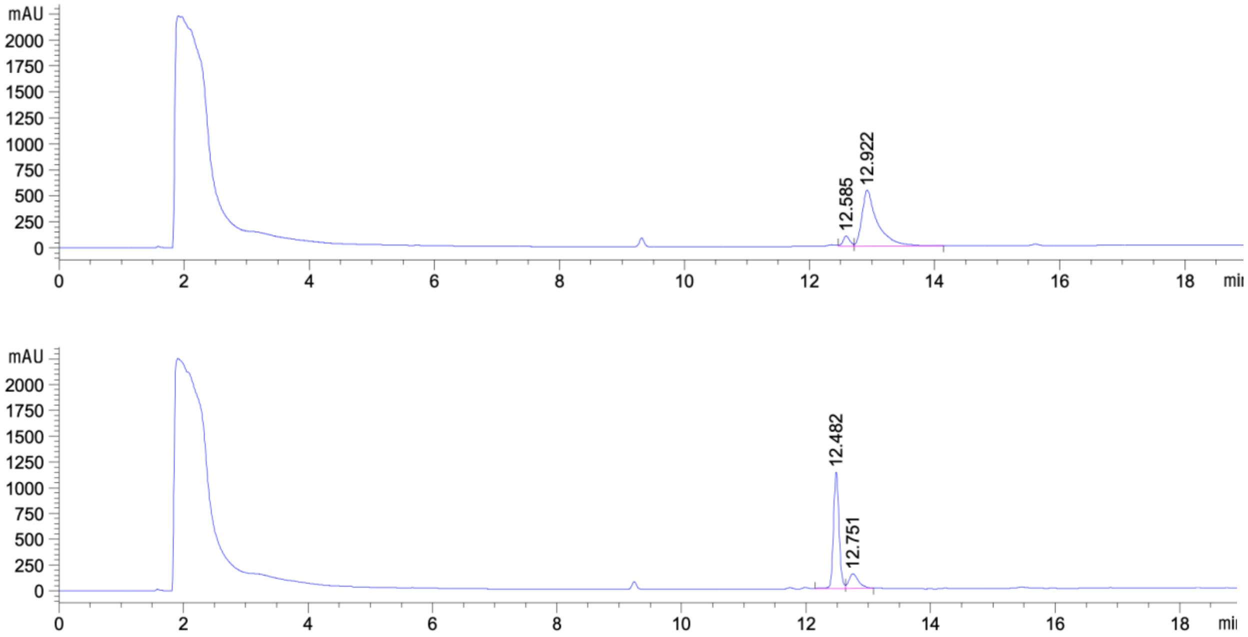

Figure S1. HPLC spectra of *trans-cis* isomerisation of **SP1** at rt in 500 μM DMSO, recorded upon 30 min and 90 min irradiation with 415 nm and 660 nm LED lights, top to bottom. 5-95% B gradient (A: 0.05% (v/v) TFA in H_2O, B: 0.05% (v/v) TFA in MeCN) over 18 min, monitored at 220 nm.

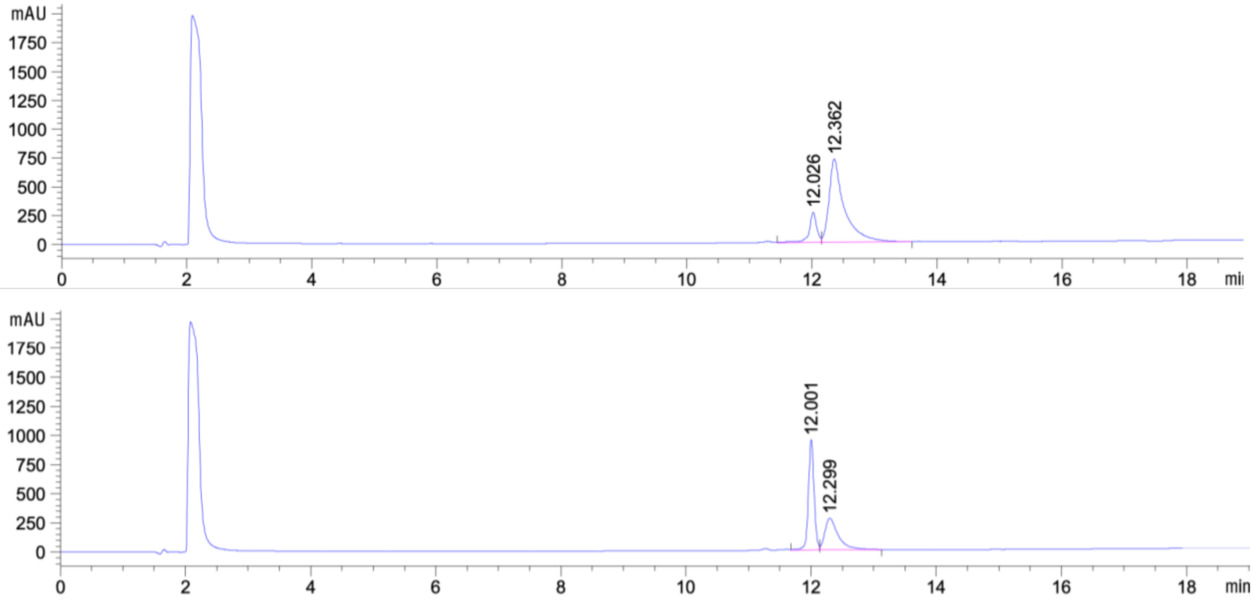

Figure S2. HPLC spectra of *trans-cis* isomerisation of **SP1** at rt in 500 μM FP assay buffer (PBS, 0.05% (v/v) Tween-20, 3% DMSO), recorded upon 30 min and 90 min irradiation with 415 nm and 660 nm LED lights, top to bottom. 5-95% B gradient (A: 0.05% (v/v) TFA in H_2O, B: 0.05% (v/v) TFA in MeCN) over 18 min, monitored at 220 nm.

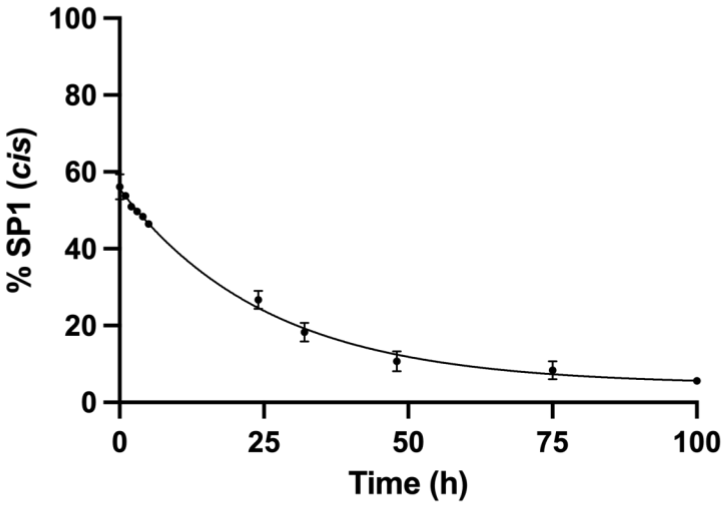

Figure S3. HPLC spectra of *cis-trans* back-isomerisation of **SP1** at rt in 500 μM FP assay buffer (PBS, 0.05% (v/v) Tween-20, 3% DMSO). 5-95% B gradient (A: 0.05% (v/v) TFA in H_2O, B: 0.05% (v/v) TFA in MeCN) over 18 min, monitored at 220 nm.

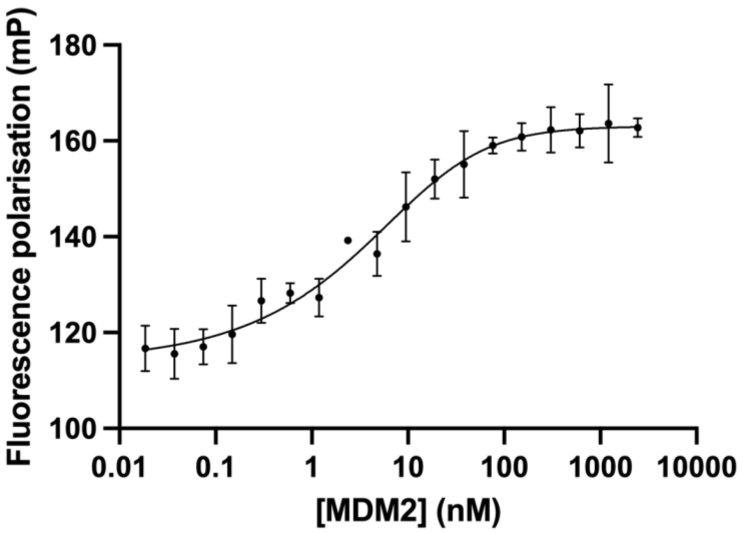

Figure S4. Direct FP inhibition assay of TAMRA-labelled peptide.

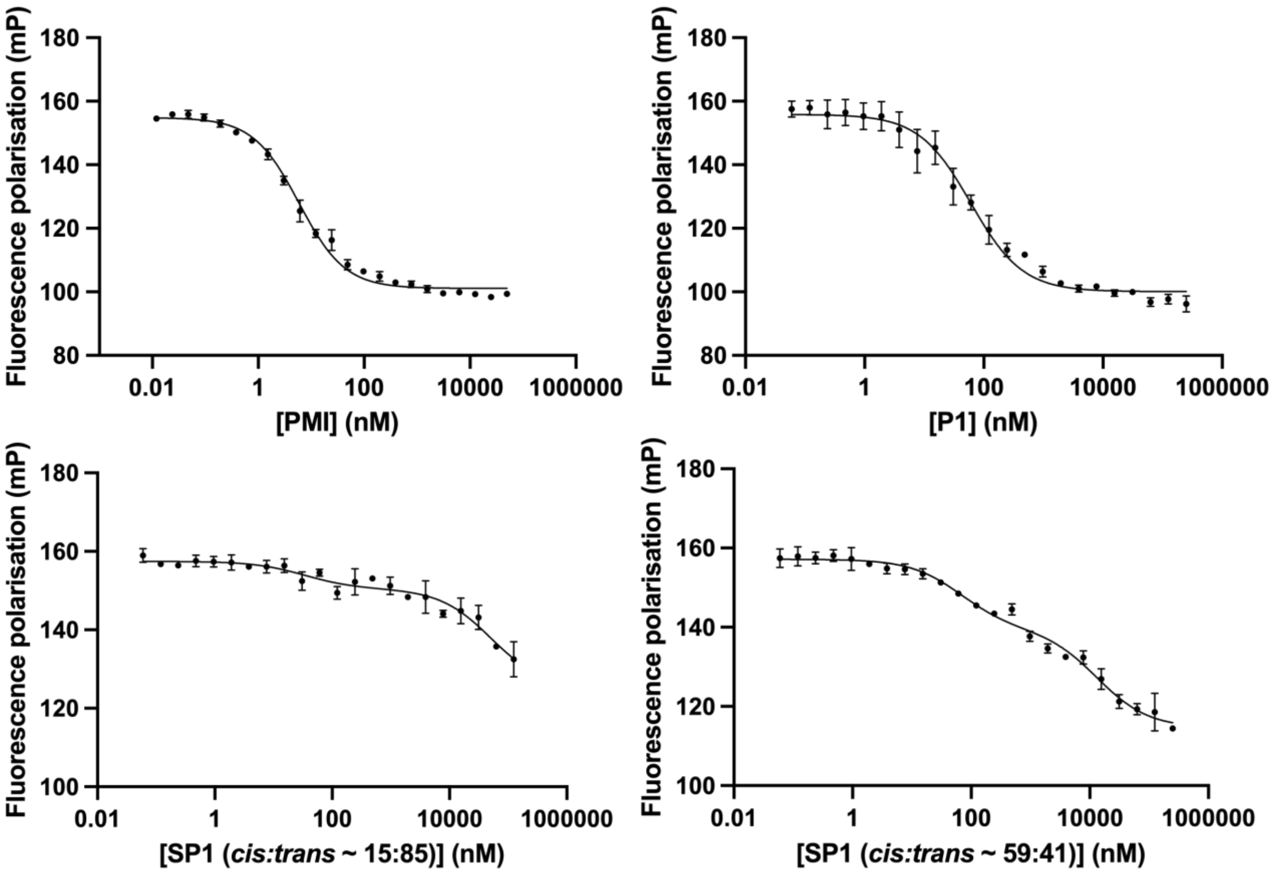

Figure S5. Competitive FP inhibition curves for peptides (top to bottom; left to right): **PMI**, **P1** and **SP1**.

Table S1. *N*-acetyl-*L*-cysteine methyl ester and *N*α-acetyl-*L*-lysine methyl ester hydrochloride conjugation with **1**, **2** and **3**, affording a range of products. Upon 2 hours, the reactions were quenched with 1N HCl and extracted with DCM. The crude product was analysed by LCMS and ^{19}F NMR analysis. *Ratio of observed products was determined by crude ^{19}F NMR analysis. **Ratio of observed products was determined by crude LCMS analysis.

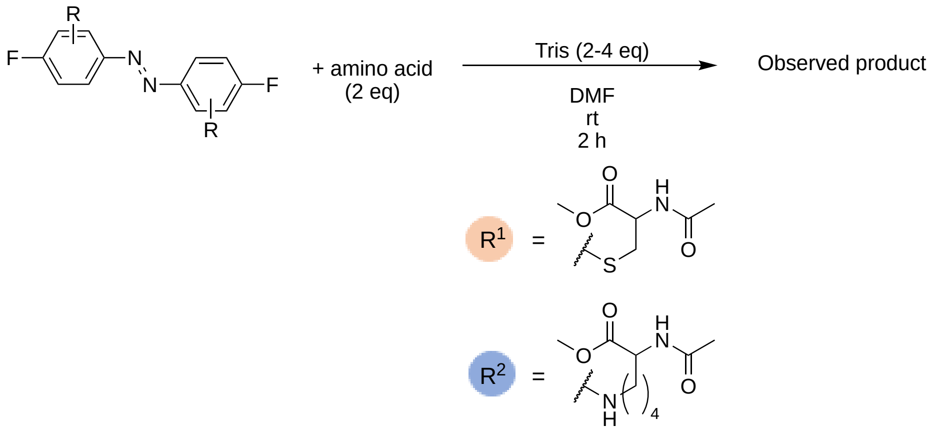

Entry	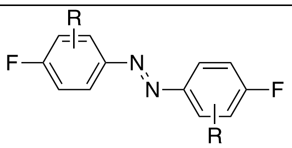	Amino Acid (eq)	Tris (eq)	Observed products

**1**	**1**	*N*-acetyl-*L*-cysteine methyl ester	2	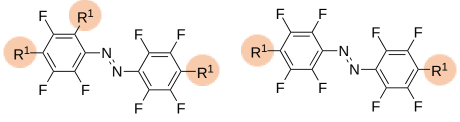 **tri**:**di**= 1:0.8*
**2**	**1**	*N*α-acetyl-*L*-lysine methyl ester hydrochloride	4	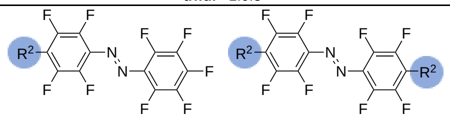 **mono**:**di** = 1:0.1*
**3**	**2**	*N*-acetyl-*L*-cysteine methyl ester	2	Mixture of mono, di and tri substituted products observed
**4**	**2**	*N*α-acetyl-*L*-lysine methyl ester hydrochloride	4	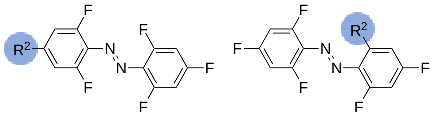
**5**	**3**	*N*-acetyl-*L*-cysteine methyl ester	2	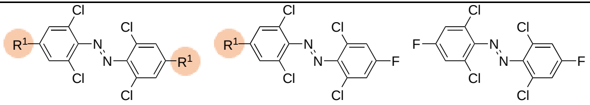 **di**:**mono**:**SM**=1:0.5:0.3*
**6**	**3**	*N*α-acetyl-*L*-lysine methyl ester hydrochloride	4	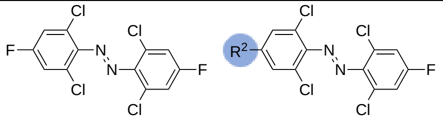 **SM**:**mono** = 94:7**

Table S2: HPLC assay stability of **PMI** in human serum.

Time (h)	Peptide Peak Area	Caffeine Peak Area	Linker/ Caffeine	Normalised Linker %
0	5941.7	9082.4	0.654	100.000
1	3933.6	6905.2	0.570	87.156
3	3720.0	7809.5	0.476	72.783
6	2067.4	6678.5	0.310	47.410
9	1351.5	8868.5	0.152	23.242
24	0.0	8995.9	0.000	0.000

Time (h)	Peptide Peak Area	Caffeine Peak Area	Linker/ Caffeine	Normalised Linker %
0	7845.5	13648.1	0.575	100.000
1	6018.9	10759.9	0.559	97.217
3	3375.0	7711.5	0.438	76.174
6	2659.3	14169.5	0.188	32.696
9	859.8	13130.6	0.065	11.304
24	0.0	16011.3	0.000	0.000

Table S3: HPLC assay stability of **P1** in human serum without the addition of TCEP.

Time (h)	Peptide Peak Area	Caffeine Peak Area	Peptide/ Caffeine	Normalised Linker %
0	8361.4	18701.9	0.447	100.000
0.5	556.4	9783.4	0.057	12.752

1	0.0	9874.4	0.000	0.000

Time (h)	Peptide Peak Area	Caffeine Peak Area	Peptide/ Caffeine	Normalised Linker %
0	3431.5	8209.4	0.418	100.000
0.5	420.4	15270.7	0.028	6.699
1	0.0	15721.1	0.000	0.000

Table S4: HPLC assay stability of **SP1** in human serum.

Time (h)	Peptide Peak Area	Caffeine Peak Area	Peptide/ Caffeine	Normalised Linker %
0	577.4	941.8	0.613	100.000
2	4145.6	7613.6	0.544	88.599
24	4619.1	9077.5	0.509	82.899
72	5514.2	11351.5	0.486	79.153
120	4064.5	8036.9	0.506	82.410

Time (h)	Peptide Peak Area	Caffeine Peak Area	Peptide/ Caffeine	Normalised Linker %
0	3952.4	5932.6	0.666	100.000
2	2268.4	3823.7	0.593	89.039
24	1809.5	3752.1	0.482	72.372
72	1266.0	3549.0	0.357	53.604
120	861.5	2697.1	0.320	48.048

2 General Experimental Details

Solvents and reagents

Unless stated otherwise, all reagents were purchased, used without further purification, and handled according to COSHH regulations. All reactions were carried out using freshly distilled solvents, under an inert N_2 atmosphere, at an atmospheric pressure and room temperature, unless otherwise stated. Acetonitrile, ethyl acetate, dichloromethane and methanol were distilled from calcium hydride. Petroleum ether 40-60 refers to the petroleum fraction with a boiling point of 40 - 60 °C. Dimethylformamide used as a solvent for peptide synthesis was purchased from Doug Discovery.

Chromatography

Reaction yields of pure compounds were reported unless otherwise stated. Thin-layer chromatography was carried out using commercially available pre-prepared glass plates Merck TLC silica gel 60 F254 silica (0.2 mm) and visualised by UV irradiation (λ = 254 nm). Manual purification was carried out using Merck 9385 Kieselgel 60 SiO_2 (230-400 mesh) under compressed air. Automated purification was carried out using Combiflash Rf200 automated chromatography system with Redisep® reverse-phase C18-silica flash columns (20 – 40 µm).

Nuclear magnetic resonance (NMR) spectroscopy

NMR spectra were acquired on 400MHz Bruker Avance III spectrometer, using a QNP probe, and 500MHz Bruker Advance III spectrometer, using an HD Smart Probe or DCH cryogenically cooled probe. ^{1}H chemical shifts (δ) are quoted in ppm to the nearest 0.01 ppm, ^{13}C and ^{19}F chemical shifts (δ) are quoted in ppm to the nearest 0.1 ppm. Coupling constants (*J*) are reported in Hertz (Hz) to the nearest 0.1 Hz. Data reported includes chemical shift, multiplicity (s, singlet; d, doublet; t, triplet; q, quartet; quint, quintet; m, multiplet; app, apparent; br, broad; or a combination thereof), number of nuclei, coupling constants and assignment. Carbon assignments are supported by DEPT (Distortionless enhanced polarization transfer), COSY (Correlation spectroscopy), HMBC (Heteronuclear multiple bond coherence), or HSQC (Heteronuclear single quantum correlation) experiments. NMR spectra were processed using Topspin v. 4.1.1 (Bruker).

Infrared (IR) spectroscopy

IR spectra were recorded using Perkin Elmer Spectrum One FT-IR spectrometer. The selected absorption maxima (ν_{max}) are quoted in wavenumbers (cm^{-1}) and described as either weak (w), medium (m), strong (s), broad (br).

Liquid chromatography-mass spectrometry (LCMS)

LCMS measurements were recorded on an Waters ACQUITY H-Class UPLC with an ESCi Multi-Mode Ionisation Waters SQ Detector 2 spectrometer using an ACQUITY UPLC® CSH C18 column (2.1 mm × 50 mm, 1.7 µm, 130 Å) at 40 °C with a gradient system 5–95% B over 5 min with constant 5% C over 1 min (solvent A: 2 mM NH_4OAc in 95:5 H_2O/MeCN; solvent B: MeCN; solvent C: 2% formic acid in H_2O). The UV absorbance was recorded across 210-800 nm, at an interval of 1.2 nm. EI refers to the electrospray ionisation technique.

High-resolution mass spectrometry (HRMS)

HRMS measurements were recorded on a Waters LCT Premier Time of Flight (ToF) mass spectrometer or a Micromass Q-TOF mass spectrometer. The values are quoted within the error limits of ±5 ppm mass unit.

High performance liquid chromatography (HPLC)

Analytical HPLC measurements were recorded on an Agilent 1200 Series machine using Agilent Eclipse XDB-C18 column (150 mm x 4.6 mm, 5 µm), with a linear gradient system (solvent A: 0.05% TFA in H_2O; solvent B: 0.05% TFA in MeCN) over 15 min at a 1 mL/min flow rate. Analytical HPLC measurements were also recorded on an Agilent 1260 Infinity machine with a reversed-phase SupelcosilTM ABZ+PLUS column (150 mm × 4.6 mm, 3 µm), with a linear gradient system (solvent A: 0.05% (v/v) TFA in H2O, solvent B: 0.05% (v/v) TFA in MeCN) over 18 minutes, at a flow rate of 1 mL/min. Analytical HPLC was monitored by UV absorbance at 220, 254 and 280 nm. Preparative HPLC was carried out on an Agilent 1260 Infinity machine using a Supelcosil ABZ+PLUS column (250 mm x 21.2 mm, 5 µm), with a linear gradient system (solvent A: 0.1% TFA in H_2O; solvent B: 0.05% TFA in MeCN) over 20 minutes at a 20 mL/min flow rate. The UV absorbance was recorded at 220, 254 and 280 nm.

Ultraviolet–visible (UV-vis) spectroscopy

UV-vis spectroscopy measurements were carried out using a Perkin-Elmer Lambda 950 spectrophotometer, over a 200-700 nm range and in quartz cuvettes with a path length of 1 mm in 1:1 H_2O/MeCN or MeCN.

2.1 Synthetic Procedures

1,2-Bis(perfluorophenyl)diazene (1)

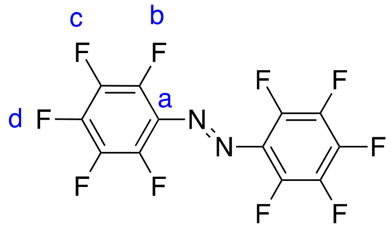

A solution of 2,3,4,5,6-perfluoroaniline (183 mg, 1.00 mmol) and DBU (300 µL, 2.00 mmol) in DCM (15.0 mL) was stirred at RT for 5 minutes under air before being cooled down to -78 °C. NCS (267 mg, 2.00 mmol) was added, and the reaction mixture was stirred for 10 minutes. The crude product was purified by silica gel chromatography (PE/EA gradient elution; product eluted with 0.1% EA in PE). **1** (68 mg, 38%) was isolated as an orange solid in a mixture of *trans*-*cis* isomers with 80:20 ratio. The compound was fully characterised as a mixture of *trans*-*cis* isomers with 73:27 ratio.

^{13}C NMR (126 MHz, $CDCl_3$) δ ppm: 143.0 (dtt, $^1J_{C-F}$ = 261.3 Hz, $^2J_{C-F}$ = 13.5 Hz, $^3J_{C-F}$ = 3.9 Hz, *trans*-C-d), 141.8 (dtt, $^1J_{C-F}$ = 259.5 Hz, $^2J_{C-F}$ = 13.6 Hz, $^3J_{C-F}$ = 4.3 Hz, *cis*-C-d), 141.5 (ddm, $^1J_{C-F}$ = 264.7 Hz, $^2J_{C-F}$ = 12.0 Hz, *trans*-C-b/c), 138.2 (dm, $^1J_{C-F}$ = 252.4 Hz, *trans*-C-b/c), 138.0 (dm, $^1J_{C-F}$ = 254.3 Hz, *cis*-C-b/c), 136.8 (dm, $^1J_{C-F}$ = 256.4 Hz, *cis*-C-b/c), 128.4 (td, $^2J_{C-F}$ = 8.3 Hz, $^4J_{C-F}$ = 4.8 Hz, *trans*-C-a).

^{19}F NMR (376 MHz, $CDCl_3$) δ ppm: -146.5 (d, *J* = 18.7 Hz, 4F, *cis*-F-b), -148.2 (d, *J* = 17.4 Hz, 4F, *trans*-F-b), -148.4 (t, *J* = 20.9 Hz, 2F, *trans*-F-d), -150.4 (t, *J* = 21.1 Hz, 2F, *cis*-F-d), -158.4-(158.6) (m, 4F, *cis*-F-c), -161.1-(-161.3) (m, 4F, *trans*-F-c).

Data in accordance with previous literature reports.[1]

1,2-Bis(2,4,6-trifluorophenyl)diazene (2)

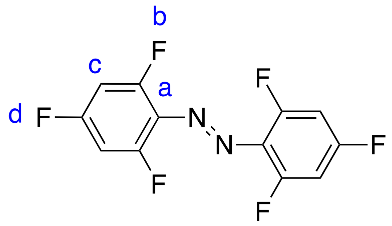

A solution of 2,4,6-trifluoroaniline (147 mg, 1.00 mmol) and DBU (300 μL, 2.00 mmol) in DCM (15.0 mL) was stirred at RT for 5 minutes under air before being cooled down to -78 °C. NCS (267 mg, 2.00 mmol) was added, and the reaction mixture was stirred for 10 minutes. The crude product was purified by silica gel chromatography (PE/EA gradient elution; product eluted with 0.1% EA in PE). **2** (60 mg, 42%) was isolated as an orange solid in a mixture of *trans-cis* isomers with 74:26 ratio. The compound was fully characterised as a mixture of *trans-cis* isomers with 86:14 ratio.

^{1}H NMR (500 MHz, $CDCl_3$) δ ppm: 6.84 (t, $^3J_{H-F}$ = 8.6 Hz, 4H, *trans*-H-c), 6.67 (t, $^3J_{H-F}$ = 8.1 Hz, 4H, *cis*-H-c).

^{13}C NMR (126 MHz, $CDCl_3$) δ ppm: 163.3 (dt, $^1J_{C-F}$ = 255.3 Hz, $^3J_{C-F}$ = 15.1 Hz, *trans*-C-d), 162.3 (dt, $^1J_{C-F}$ = 253.2 Hz, $^3J_{C-F}$ = 14.3 Hz, *cis*-C-d), 156.6 (ddd, $^1J_{C-F}$ = 266.9 Hz, $^3J_{C-F}$ = 15.2, 6.7 Hz, *trans*-C-b), 152.3 (ddd, $^1J_{C-F}$ = 251.1 Hz, $^3J_{C-F}$ = 14.8, 8.1 Hz, *cis*-C-b), 129.0 (td, $^2J_{C-F}$ = 9.6, $^4J_{C-F}$ = 5.0 Hz, *trans*-C-a), 101.7 (ddd, $^2J_{C-F}$ = 26.2, 24.6 Hz, $^4J_{C-F}$ = 3.9 Hz, *trans*-C-c), overlapped 101.3 (dd, $^2J_{C-F}$ = 24.9 Hz, $^4J_{C-F}$ = 3.3 Hz, *cis*-C-c).

^{19}F NMR (471 MHz, $CDCl_3$) δ ppm: -103.6 (t, *J* = 8.3 Hz, 2F, *trans*-F-d), -106.4-(-106.5) (m, 2F, *cis*-F-d), -116.7-(-116.8) (m, 4F, *cis*-F-b), -117.4 (d, *J* = 8.4 Hz, 4F, *trans*-F-b).

Data in accordance with previous literature reports.[1]

1,2-Bis(2,6-dichloro-4-fluorophenyl)diazene (3)

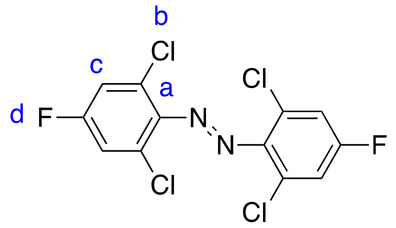

A solution of 2,6-dichloro-4-fluoroaniline (1.00 g, 5.96 mmol) and DBU (1.70 mL, 11.1 mmol) in DCM (90 mL) was stirred at RT for 5 minutes under air before being cooled down to -78 °C. NCS (1.49 g, 11.1 mmol) was added, and the reaction mixture was stirred for 10 minutes. Upon completion, the solution was quenched with $NaHCO_3$ (30 mL) and extracted with DCM (3 x 50 mL). The combined organic fractions were washed with H_2O (50 mL) and 1N HCl (50 mL), dried ($NaSO_4$), filtered and concentrated *in vacuo* to yield the crude product. The crude product was purified by silica gel chromatography (100% PE elution). **3** (236 mg, 24%) was isolated as an orange solid in a mixture of *trans*-*cis* isomers with 87:13 ratio. The compound was fully characterised as a mixture of *trans*-*cis* isomers with 87:13 ratio.

^{1}H NMR (400 MHz, $CDCl_3$) δ ppm: 7.26 (d, J = 7.9 Hz, 4H, *trans*-H-c), 7.08 (d, J = 7.8 Hz, 4H, *cis*-H-c).

^{13}C NMR (126 MHz, $CDCl_3$) δ ppm: 161.3 (d, $^1J_{C-F}$ = 255.4 Hz, *trans*-C-d), 161.2 (d, $^1J_{C-F}$ = 255.2 Hz, *trans*-C-d), 144.9 (d, $^4J_{C-F}$ = 4.3 Hz, *cis*-C-a), 144.3 (d, $^4J_{C-F}$ = 4.2 Hz, *trans*-C-a), 129.1 (d, $^3J_{C-F}$ = 11.8 Hz, *trans*-C-b), 127.2 (d, $^3J_{C-F}$ = 11.7 Hz, *cis*-C-b), 117.3 (d, $^2J_{C-F}$ = 25.2 Hz, *trans*-C-c), 117.1 (d, $^2J_{C-F}$ = 25.2 Hz, *cis*-C-c).

^{19}F NMR (376 MHz, $CDCl_3$) δ ppm: -110.1 (s, 2F, *trans*-F-d), -110.7 (s, 2F, *cis*-F-d).

IR (ATR) ν_{max} (neat/cm^{-1}): 3340 (m, N=N), 1500 (C=C, s), 1396 (m), 1393 (s), 1243 (m), 1191 (m), 951 (m), 804 (m), 623 (m).

Dimethyl 3,3'-((diazene-1,2-diylbis(3,5-dichloro-4,1-phenylene))bis(sulfanediyl))-bis(2-acetamidopropanoate) (4)

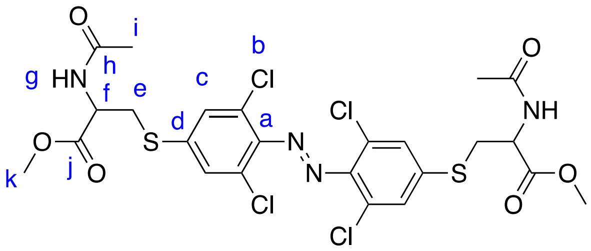

A solution of **3** (56.7 mg, 159 µmol), *N*-acetyl-L-cysteine methyl ester (169.1 mg, 956 µmol) and TRIS base (115.6 mg, 956 µmol) in DMF (16 mL) was stirred at RT for 4.5 hours. The crude product was evaporated *in vacuo* and subsequently purified by reverse-phase flash column chromatography (5-100% solvent B in solvent A; Solvent A: 0.1 M NH_4OH (aq), Solvent B: MeCN) and lyophilised to yield the product **4** (57.0 mg, 53%) was afforded as an orange solid, in its *trans* isomer form.

^{1}H NMR (400 MHz, DMSO-d_6) δ ppm: 8.55 (d, *J* = 7.8 Hz, 2H, *trans*-H-g), 7.66 (s, 4H, *trans*-H-c), 4.53 (dt, *J* = 7.9, 5.2 Hz, 2H, *trans*-H-f), 3.56 (s, 6H, *trans*-H-k), 3.62-3.40 (m, 4H, *trans*-H-e), 1.85 (s, 6H, *trans*-H-i).

^{13}C NMR (126 MHz, $CDCl_3$) δ ppm: 170.6 (*trans*-C-j), 169.6 (*trans*-C-h), 143.7 (*trans*-C-b), 140.8 (trans-C-d), 127.9 (*trans*-C-c), 127.2 (trans-C-a), 52.3 (trans-C-k), 51.3 (trans-C-f), 33.1 (*trans*-C-e), 22.2 (*trans*-C-i).

HRMS (ESI): $[M+H]^+$ calc. for $C_{24}H_{24}Cl_4N_4O_6S_2$: 668.9973 observed 668.9962.

Tetramethyl 3,3',3'',3'''-((diazene-1,2-diylbis(2,5,6-trifluorobenzene-4,1,3-triyl))tetrakis(sulfanediyl))-tetrakis(2-acetamidopropanoate) (5a)

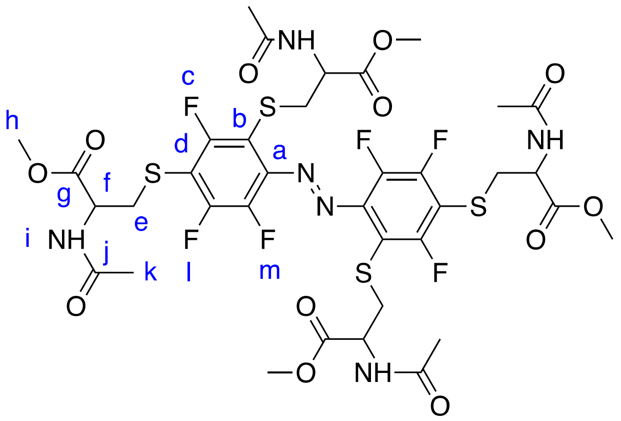

A solution of **1** (50.0 mg, 138 µmol), *N*-acetyl-L-cysteine methyl ester (122.0 mg, 690 µmol) and TRIS base (83.6 mg, 690 µmol) in DMF (14 mL) was stirred at RT for 2 hours. The crude product was evaporated *in vacuo* and subsequently purified by silica gel chromatography (DCM/MeOH gradient elution; product eluted with 7% MeOH in DCM). **5a** (60.0 mg, 44%) was afforded as a red oil, in its *trans* isomer form.

^{1}H NMR (400 MHz, $CDCl_3$) δ ppm: 6.67 (d, *J* = 6.9 Hz, 2H, H-i), 6.61 (d, *J* = 6.4 Hz, 2H, H-i), overlapped 4.84 (dt, *J* = 7.0, 5.1 Hz, 2H, H-f), overlapped 4.80 (dt, *J* = 7.5, 4.6 Hz, 2H, H-f), 3.72 (s, 6H, H-h), 3.63 (s, 6H, H-h), 3.56-3.33 (m, 8H, H-e), 2.02 (s, 6H, H-h), 1.92 (s, 6H, H-h).

^{13}C NMR (101 MHz, $CDCl_3$) δ ppm: 170.7 (C-g), 170.5 (C-g), 170.3 (C-j), 170.2 (C-j), 158.5 (app d, $^1J_{C\text{-}F}$ = 244.5 Hz, C-c), 151.0 (ddd, $^1J_{C\text{-}F}$ = 249.9 Hz, $^2J_{C\text{-}F}$ = 12.5 Hz, $^3J_{C\text{-}F}$ = 5.2 Hz, C-l), 143.1 (C-a), 139.0 (ddd, $^1J_{C\text{-}F}$ = 266.1 Hz, $^2J_{C\text{-}F}$ = 15.7 Hz, $^4J_{C\text{-}F}$ = 4.3 Hz, C-m), 115.4-114.9 (m, C-b, C-d), 53.2 (C-h), 53.0 (C-h), 52.6 (C-f), 52.5 (C-f), 37.1 (d, *J* = 4.9 Hz, C-e), 36.0 (app s, C-e), 23.0 (C-k), 22.6 (C-k).

^{19}F NMR (376 MHz, $CDCl_3$) δ ppm: -100.1 (d, *J* = 13.3 Hz, F-c), -125.7 (d, *J* = 21.9 Hz, F-m), -152.1 (dd, *J* = 21.7, 13.5 Hz, F-l).

IR (ATR) ν_{max} (neat/cm^{-1}): 3295 (m, N=N), 2957 (m, CH), 1744 (s, C=O, ester), 1654 (s, C=O, amide), 1537 (s), 1531 (s), 1452 (s), 1437 (s), 1372 (s), 1322 (s), 1276 (s), 1221 (s), 1172 (s), 1074 (w), 1009 (w), 915 (s), 858 (w), 832 (w), 786 (w), 685 (w), 685 (w).

HRMS (ESI): $[M+H]^+$ calc. for $C_{36}H_{40}F_6N_6O_{12}S_4$: 991.1569 observed 991.1575.

Trimethyl 3,3',3''-((2-((2,4-bis((2-acetamido-3-methoxy-3-oxopropyl)thio)-3,5,6-trifluorophenyl)diazenyl)-4,6-difluorobenzene-1,3,5-triyl)tris(sulfanediyl))-tris(2-acetamidopropanoate) (5b)

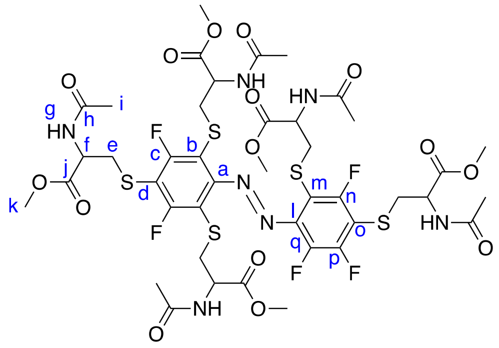

A solution of **1** (50.0 mg, 138 µmol), *N*-acetyl-L-cysteine methyl ester (122.0 mg, 690 µmol) and TRIS base (83.6 mg, 690 µmol) in DMF (14 mL) was stirred at RT for 2 hours. The crude product was evaporated *in vacuo* and subsequently purified by silica gel chromatography (DCM/MeOH gradient elution; product eluted with 7% MeOH in DCM). **5b** (60.0 mg, 38%) was afforded as a red oil, in its *trans* isomer form. Chemical shifts and coupling pattern in ^{13}C and ^{19}F NMR spectra compared to that of compound **5a** and used for assignment of the l-q carbon/fluorine signals.

^{1}H NMR (400 MHz, $CDCl_3$) δ ppm: 6.97 (d, *J* = 7.4 Hz, 1H, H-g), 6.82 (d, *J* = 7.4 Hz, 1H, H-g), 6.78 (d, *J* = 7.4 Hz, 2H, H-g), 6.74 (d, *J* = 7.3 Hz, 1H, H-g), 4.85-4.65 (m, 5H, H-f), 3.72 (s, 3H, H-k), 3.71 (s, 3H, H-k), 3.63 (s, 3H, H-k), 3.61 (s, 6H, H-k), 3.57-3.34 (m, 8H, H-e), 3.25 (dd, *J* = 13.9, 5.4 Hz, 2H, H-e), 2.15 (s, 3H, H-i), 2.00 (s, 3H, H-i), 1.99 (s, 3H, H-i), overlapped 1.89-1.88 (m, 6H, H-i).

^{13}C NMR (126 MHz, $CDCl_3$) δ ppm: 170.71 (C-j), 170.68 (2C, C-j), 170.54 (C-j), 170.45 (C-j), 170.39 (C-h), 170.36 (C-h), 170.30 (C-h), 170.26 (2C, C-h), 162.6 (dd, $^{1}J_{C-F}$ = 248.6 Hz, $^{3}J_{C-F}$ = 6.0 Hz, C-c), 158.4 (dm, $^{1}J_{C-F}$ = 244.1 Hz, C-n), 156.9 (t, $^{3}J_{C-F}$ = 3.0 Hz, C-a), 150.9 (ddd, $^{1}J_{C-F}$ = 251.3 Hz, $^{2}J_{C-F}$ = 13.3 Hz, $^{3}J_{C-F}$ = 5.7 Hz, C-p), 142.0 (dd, $^{3}J_{C-F}$ = 5.4, 2.0 Hz, C-l), 138.8 (dm, $^{1}J_{C-F}$ = 265.9 Hz, C-q), 116.5 (dd, $^{2}J_{C-F}$ = 23.3 Hz, $^{3}J_{C-F}$ = 4.0 Hz, C-m/o), 115.2 (dd, $^{2}J_{C-F}$ = 26.5, 18.9 Hz, C-d), 112.9 (dd, $^{2}J_{C-F}$ = 27.1 Hz, $^{3}J_{C-F}$ = 7.5 Hz, C-b), overlapped 112.8 (m, C-m/o), 52.99 (C-k), 52.92 (C-k), 52.88 (C-k), 52.82 (2C, C-k), 52.73 (C-k), 52.66 (C-k), 52.5 (C-k), 52.4 (2C, C-k), 37.2-37.1 (m, 2C, C-e), 37.07 (d, J = 5.5 Hz, 2C, C-e), 35.9 (d, *J* = 19.4 Hz, 2C, C-e), 22.9 (d, *J* = 0.8 Hz, 2C, C-i), 22.80 (C-i), 22.76 (m, 2C, C-i).

^{19}F NMR (376 MHz, $CDCl_3$) δ ppm: -93.0 (s, 2F, F-c), -100.3 (d, *J* = 13.0 Hz, 1F, F-n), -125.9 (d, *J* = 22.4 Hz, 1F, F-q), -152.1 (dd, *J* = 21.8, 13.7 Hz, 1F, F-p).

IR (ATR) ν_{max} (neat/cm^{-1}): 3288 (w, N=N), 3059 (w, N=N), 2250 (w, C-H), 1740 (s, C=O, ester), 1654 (s, C=O, amide), 1552 (s), 1435 (s), 1372 (s), 1341 (s), 1308 (s), 1213 (s), 1175 (s), 1130 (m), 1037 (m), 1008 (m), 983 (m), 907 (s), 838 (m), 735 (s).

HRMS (ESI): $[M+Na]^+$ calc. for $C_{42}H_{50}F_5N_7O_{15}S_5$ 1170.1781 observed 1170.1759.

2.2 Cysteine and Lysine Reactivity Assessment

1, LCMS

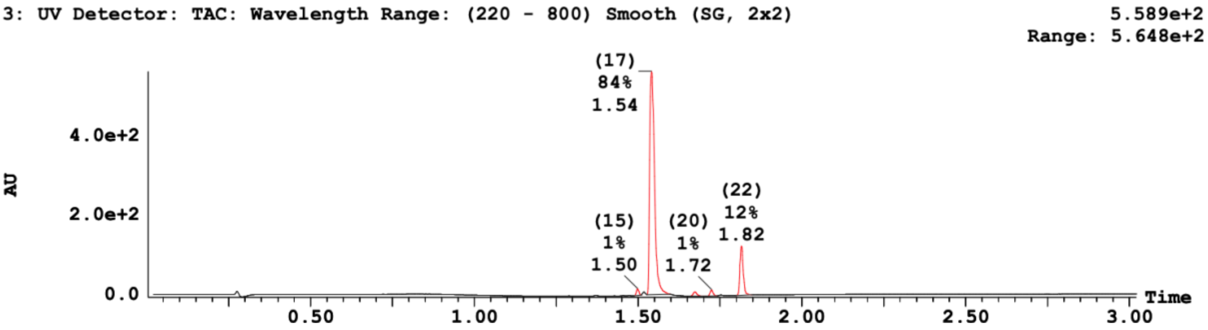

1 (1 eq) + *N*-acetyl-L-cysteine methyl ester (2 eq) + Tris (2 eq), 2h, rt, LCMS

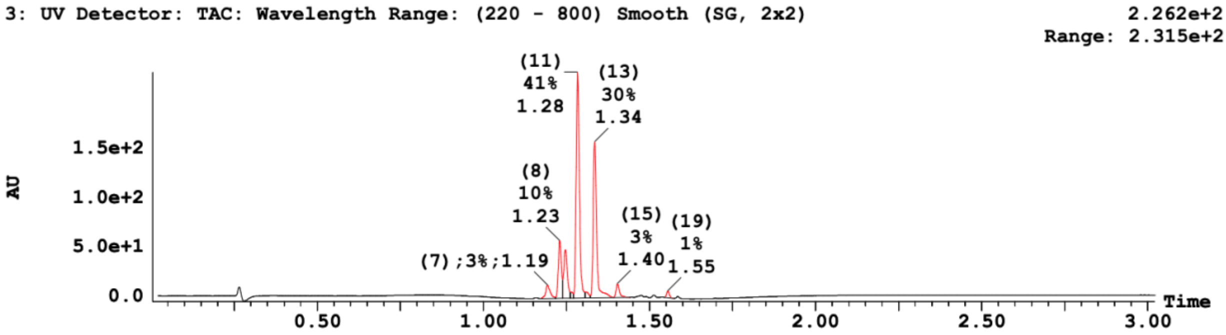

Peak ID	Compound	Time	Mass Found	BPM
11		1.28	Not Found	834

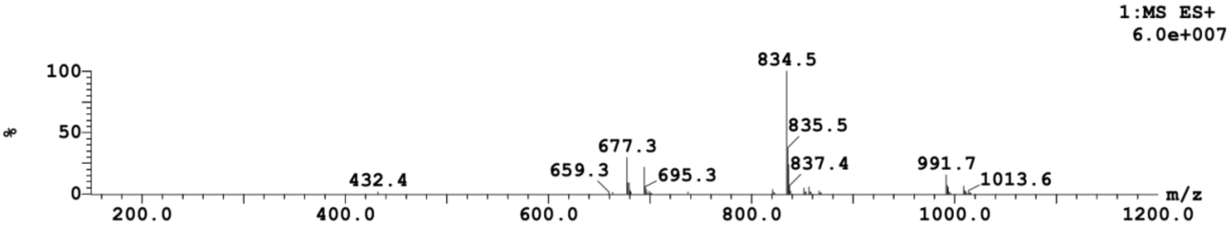

Peak ID	Compound	Time	Mass Found	BPM
13		1.34	Not Found	834

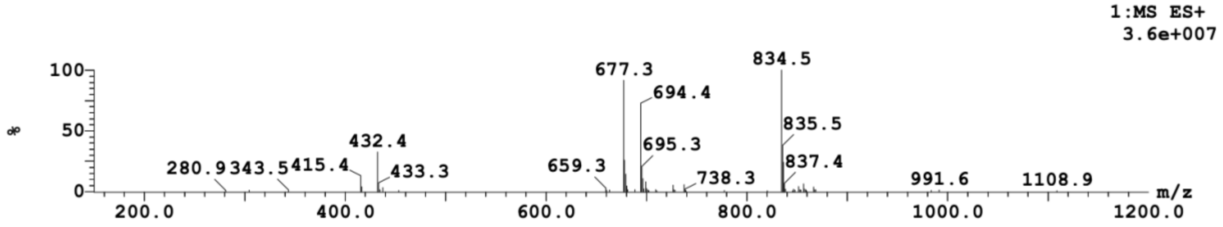

1 (1 eq) + *N*-acetyl-L-cysteine methyl ester (2 eq) + Tris (4 eq), 2h, rt, ^{19}F NMR

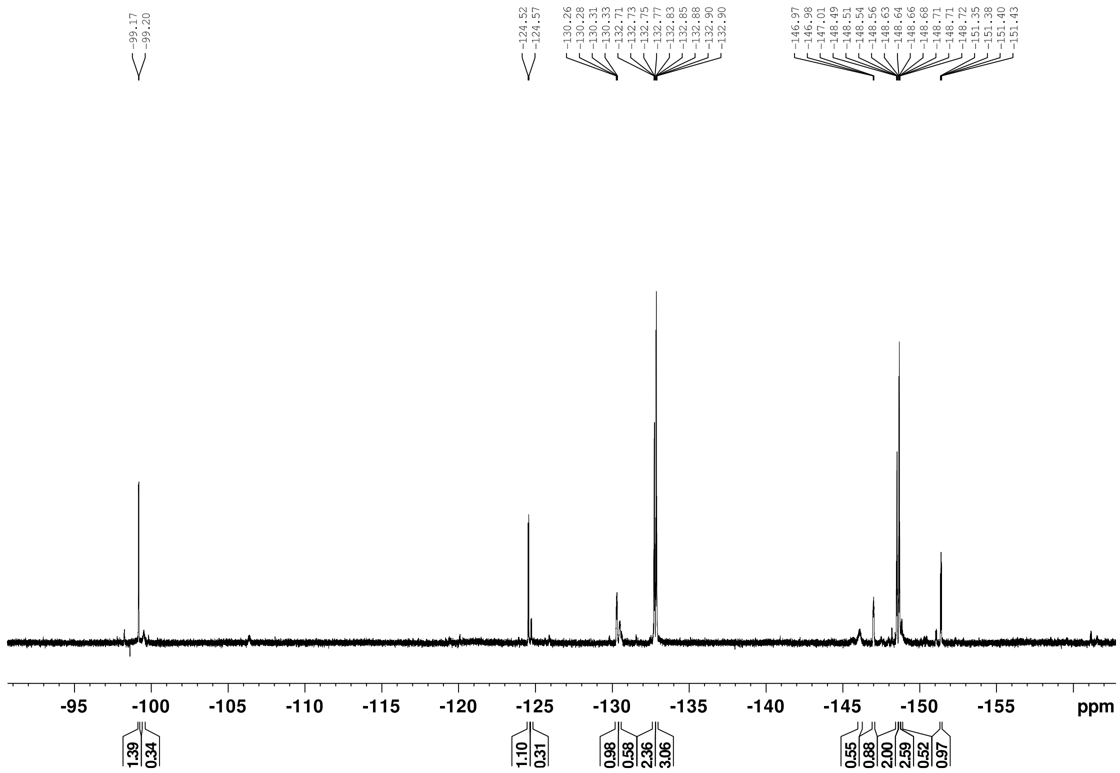

1 (1 eq) + *N*α-acetyl-*L*-lysine methyl ester hydrochloride (2 eq) + Tris (4 eq), 2h, rt, LCMS

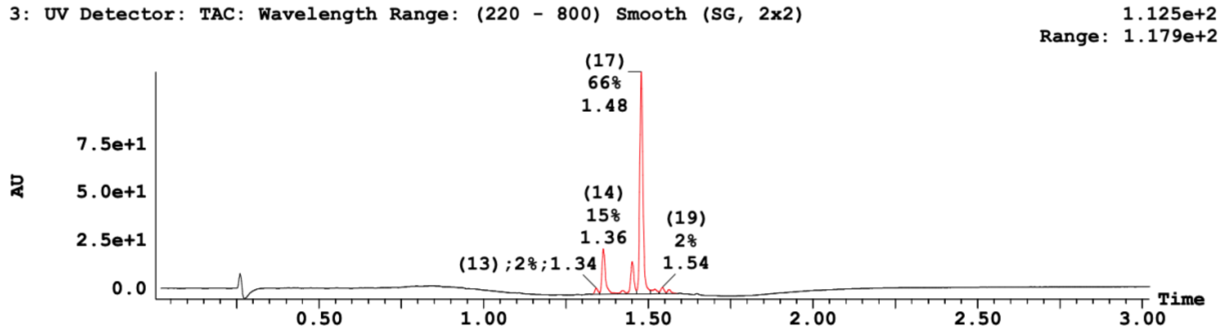

Peak ID	Compound	Time	Mass Found	BPM
14		1.36	Not Found	728

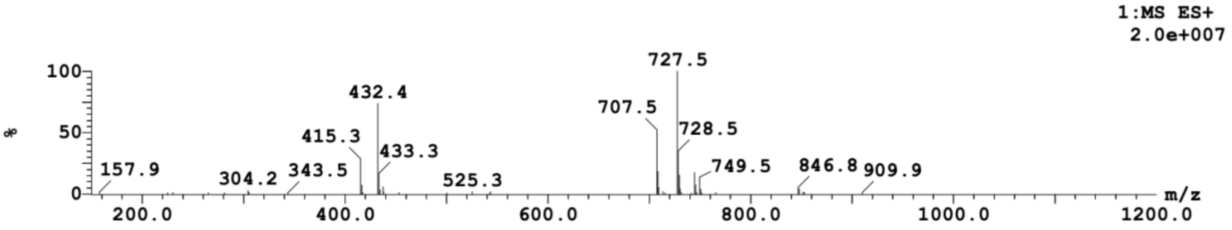

Peak ID	Compound	Time	Mass Found	BPM
17		1.48	Not Found	545

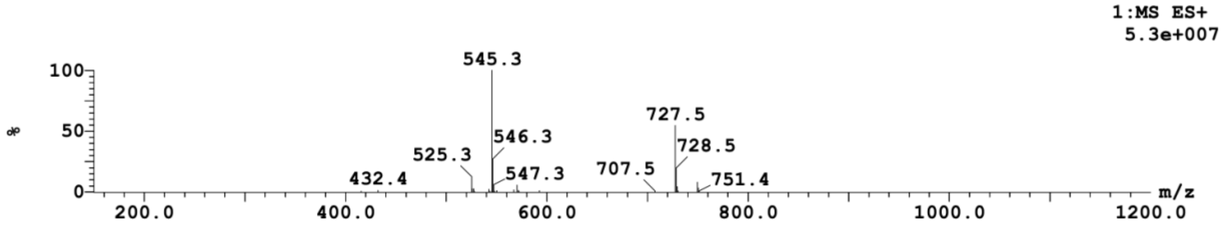

1 (1 eq) + *N*α-acetyl-*L*-lysine methyl ester hydrochloride (2 eq) + Tris (4 eq), 2h, rt, ^{19}F NMR

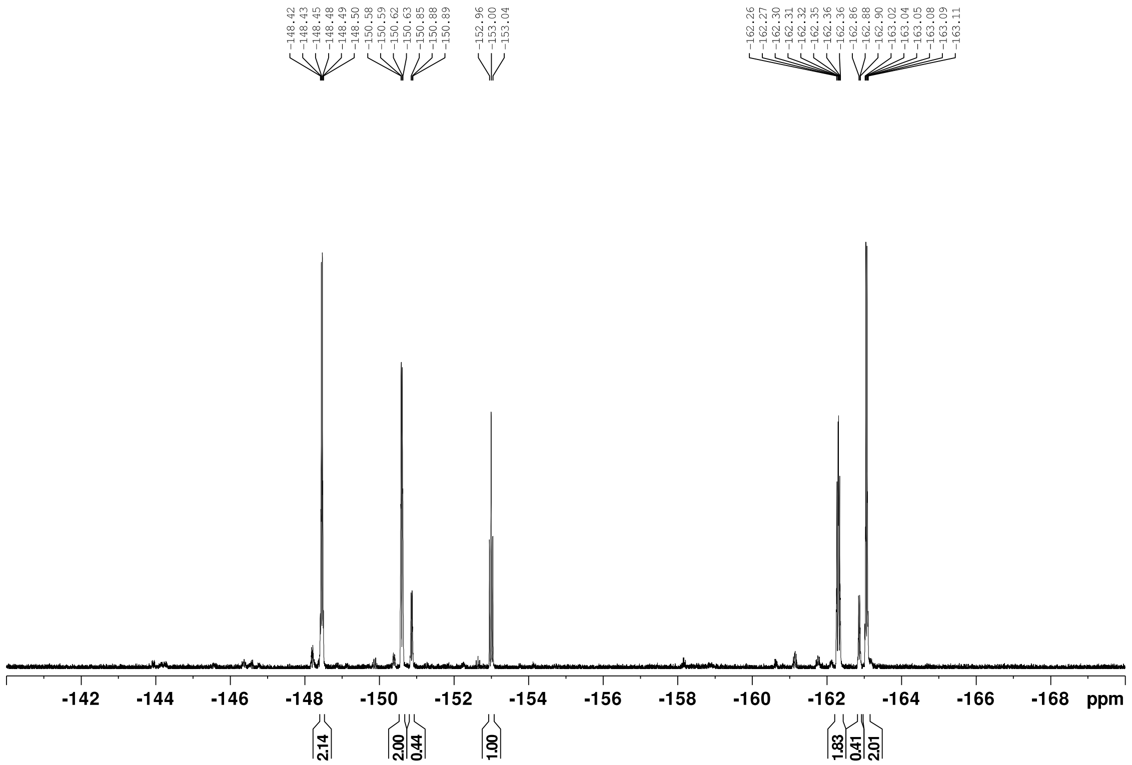

2, LCMS

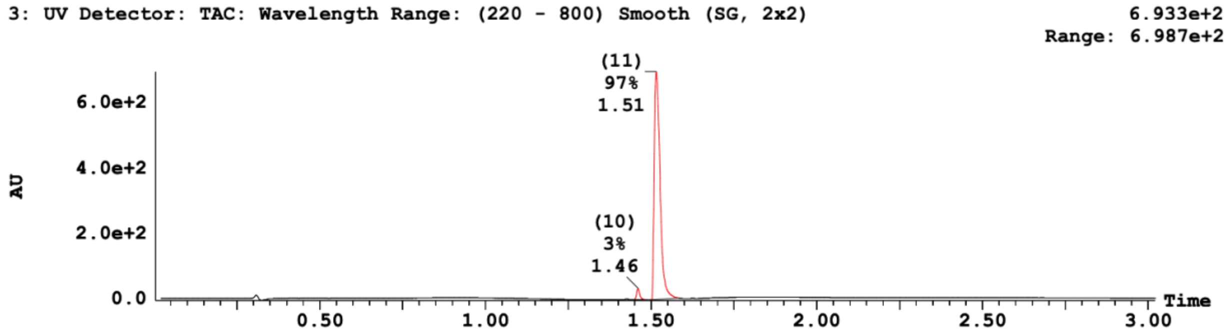

2 (1 eq) + *N*-acetyl-L-cysteine methyl ester (2 eq) + Tris (2 eq), 2h, rt, LCMS

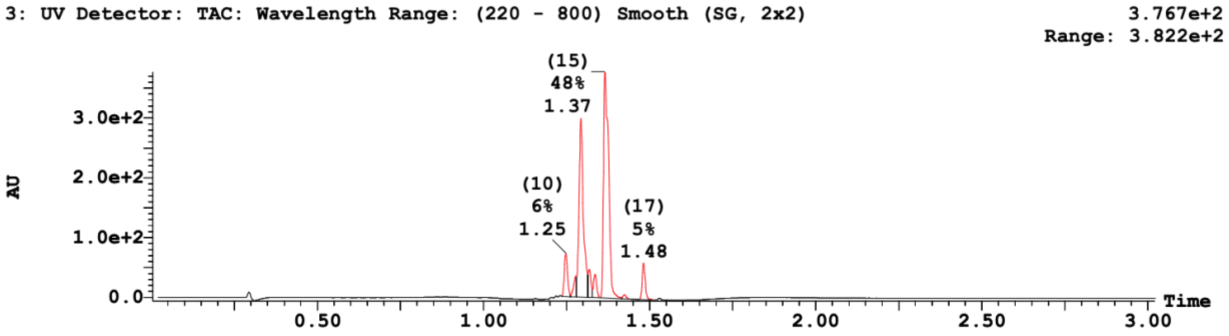

Peak ID	Compound	Time	Mass Found	BPM
10		1.25	Not Found	605

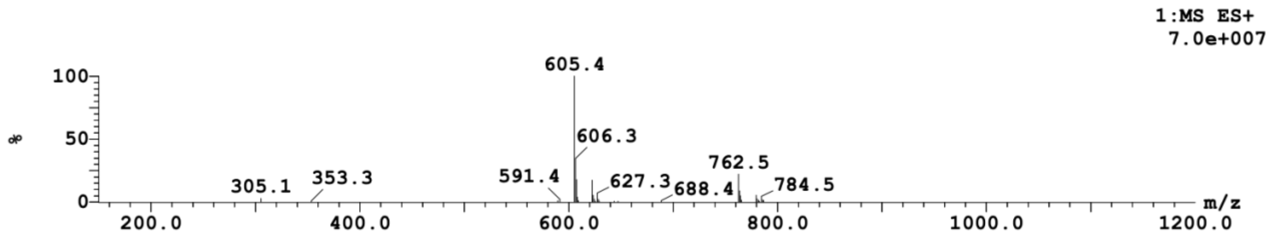

Peak ID	Compound	Time	Mass Found	BPM
12		1.29	Not Found	649

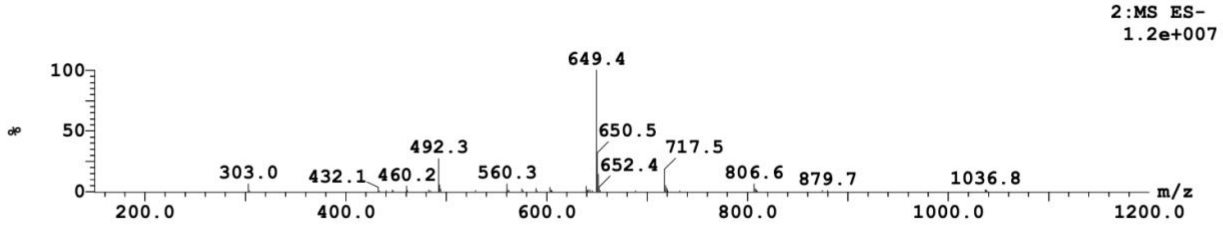

Peak ID	Compound	Time	Mass Found	BPM
14		1.33	Not Found	649

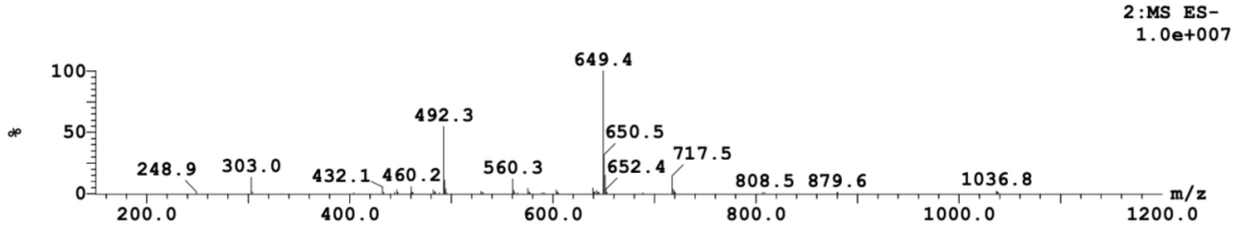

Peak ID	Compound	Time	Mass Found	BPM
18		1.53	Not Found	448

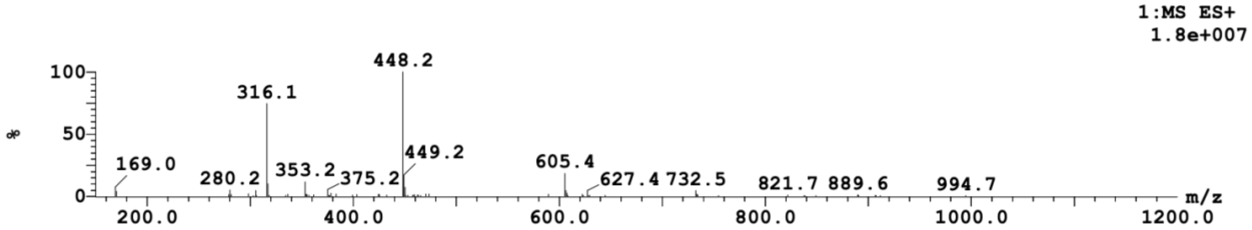

2 (1 eq) + *N*-acetyl-L-cysteine methyl ester (2 eq) + Tris (4 eq), 2h, rt, ^{19}F NMR

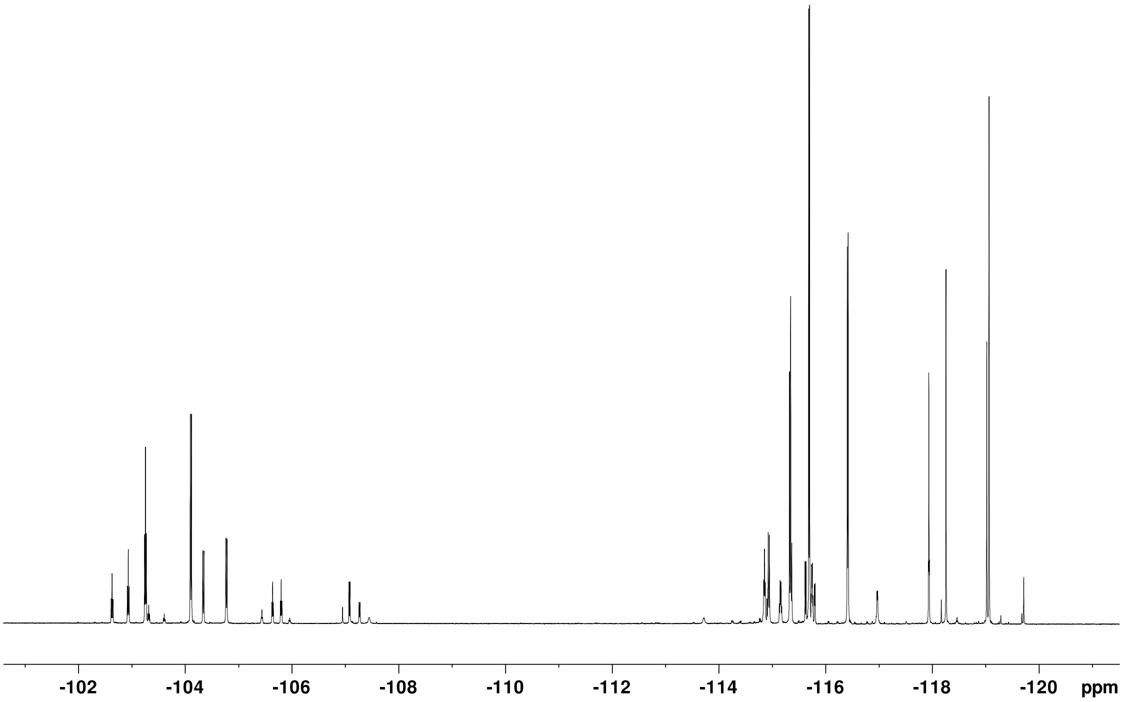

2 (1 eq) + *N*α-acetyl-*L*-lysine methyl ester hydrochloride (2 eq) + Tris (4 eq), 2h, rt, LCMS

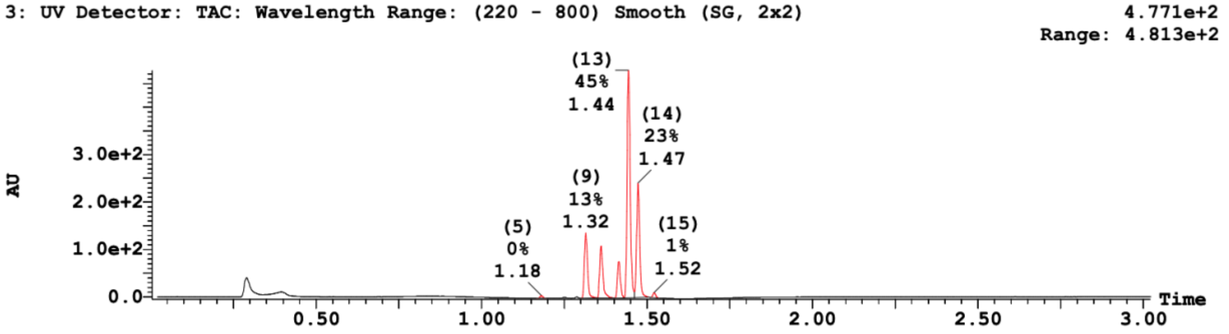

Peak ID	Compound	Time	Mass Found	BPM
9		1.32	Not Found	453

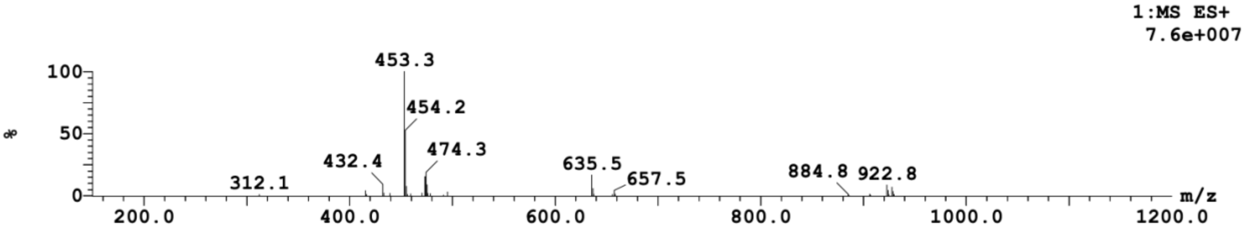

Peak ID	Compound	Time	Mass Found	BPM
11		1.36	Not Found	453

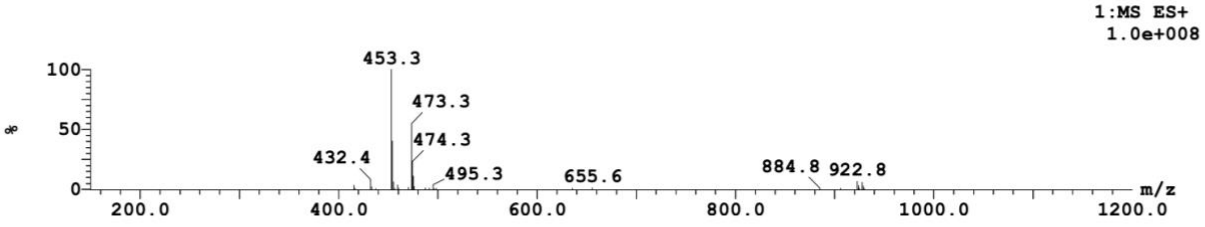

Peak ID	Compound	Time	Mass Found	BPM
13		1.46	Not Found	473

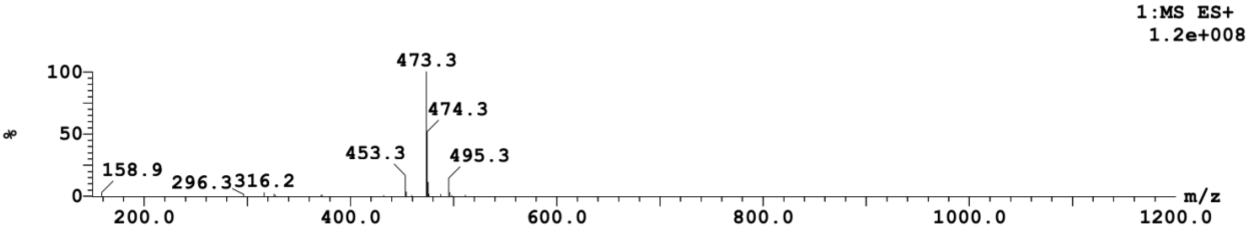

2 (1 eq) + *N*α-acetyl-*L*-lysine methyl ester hydrochloride (2 eq) + Tris (4 eq), 2h, rt, ^{19}F NMR

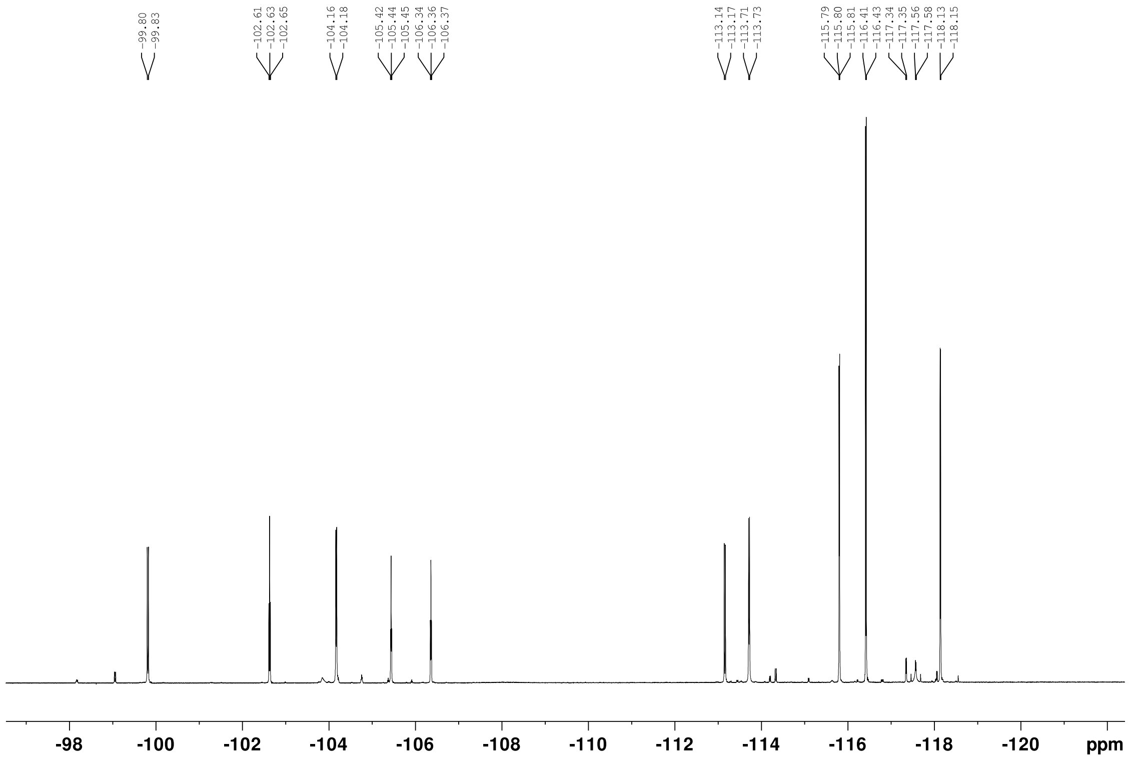

3, LCMS

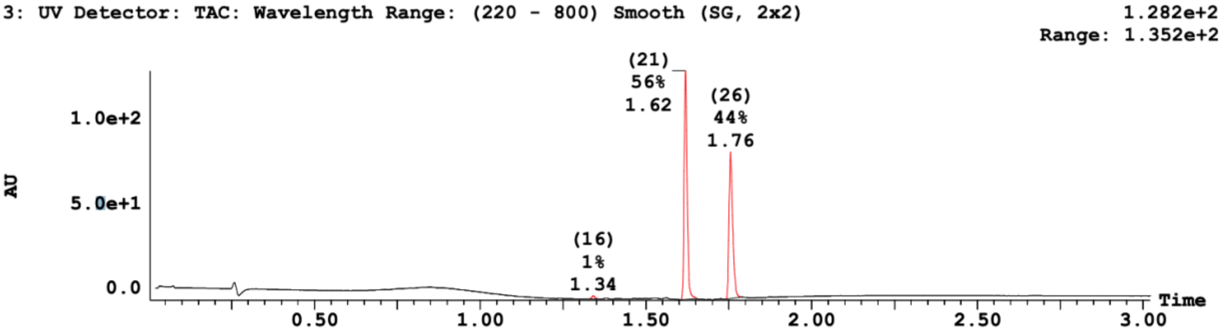

3 (1 eq) + *N*-acetyl-L-cysteine methyl ester (2 eq) + Tris (2 eq), 2h, rt, LCMS

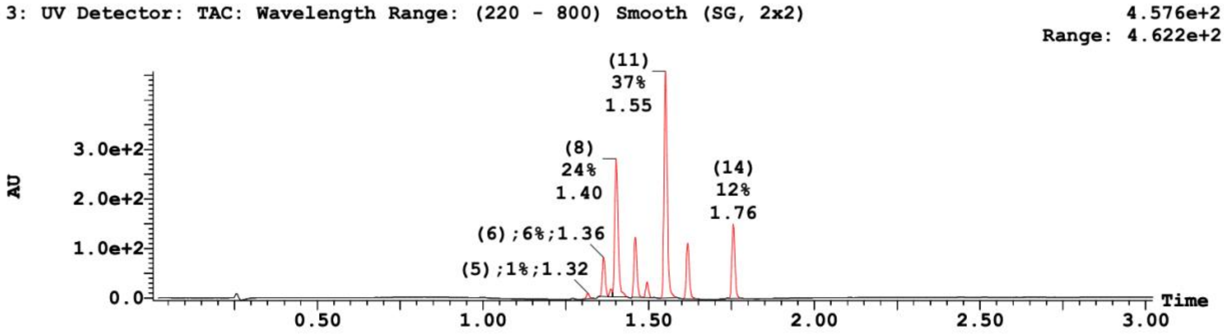

Peak ID	Compound	Time	Mass Found	BPM
6		1.37	Not Found	671

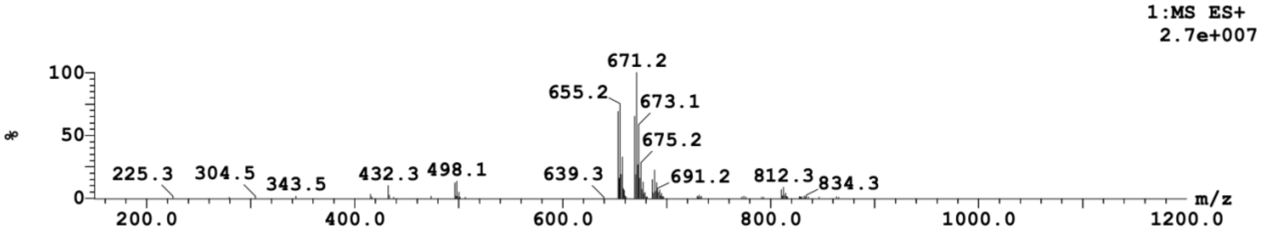

Peak ID	Compound	Time	Mass Found	BPM
8		1.42	Not Found	671

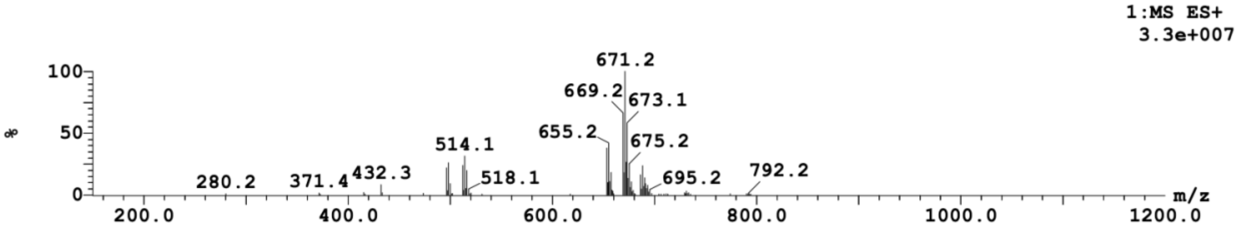

Peak ID	Compound	Time	Mass Found	BPM
9		1.48	Not Found	558

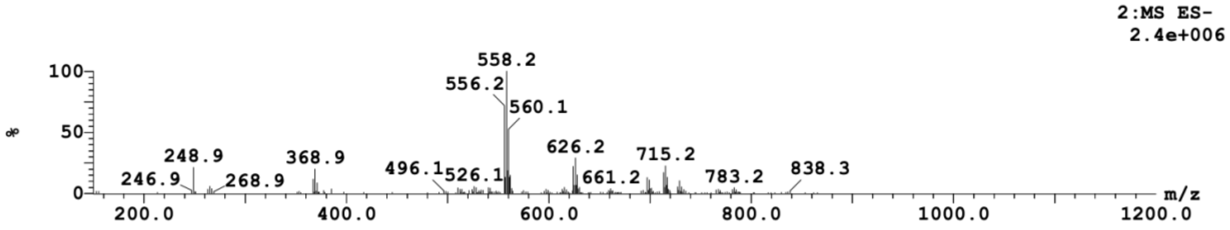

Peak ID	Compound	Time	Mass Found	BPM
11		1.55	Not Found	514

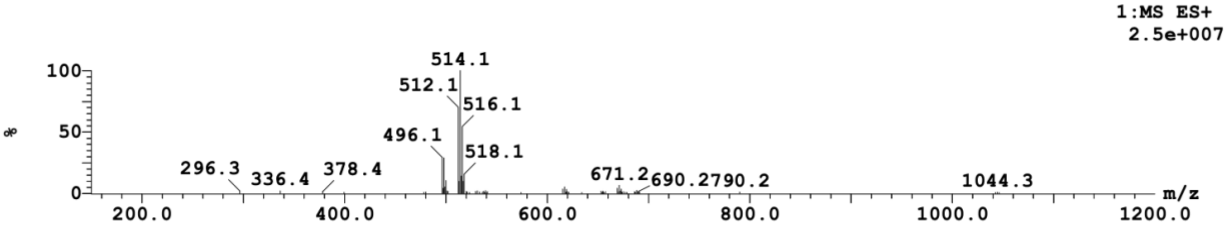

3 (1 eq) + *N*-acetyl-L-cysteine methyl ester (2 eq) + Tris (4 eq), 2h, rt, ^{19}F NMR

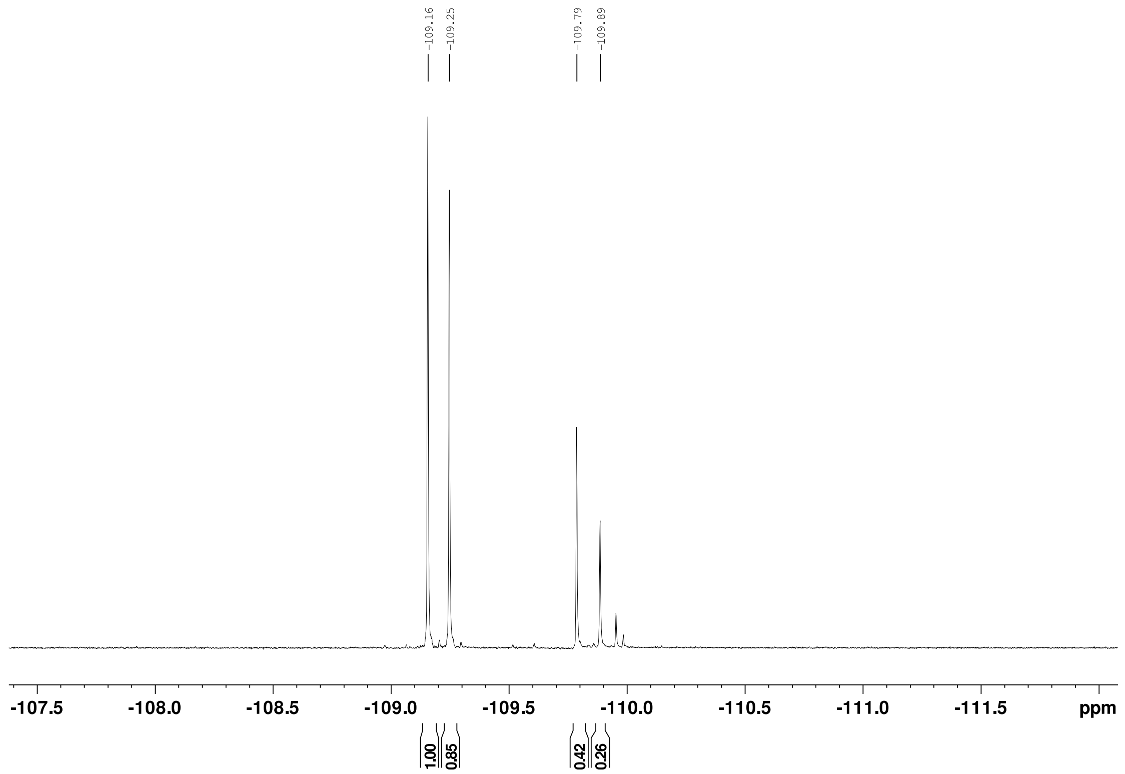

3 (1 eq) + *N*-acetyl-L-cysteine methyl ester (2 eq) + Tris (4 eq), 2h, rt, ^{1}H (aromatic) NMR

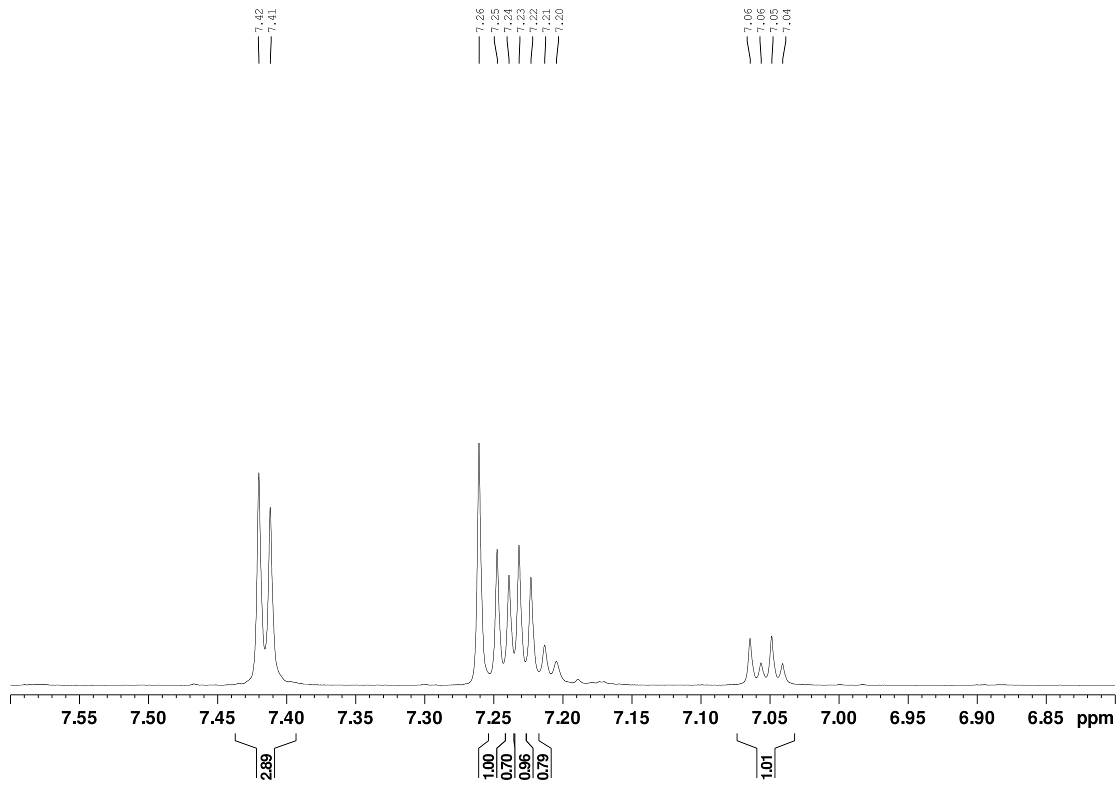

3 (1 eq) + *N*α-acetyl-*L*-lysine methyl ester hydrochloride (2 eq) + Tris (4 eq), 2h, rt, LCMS

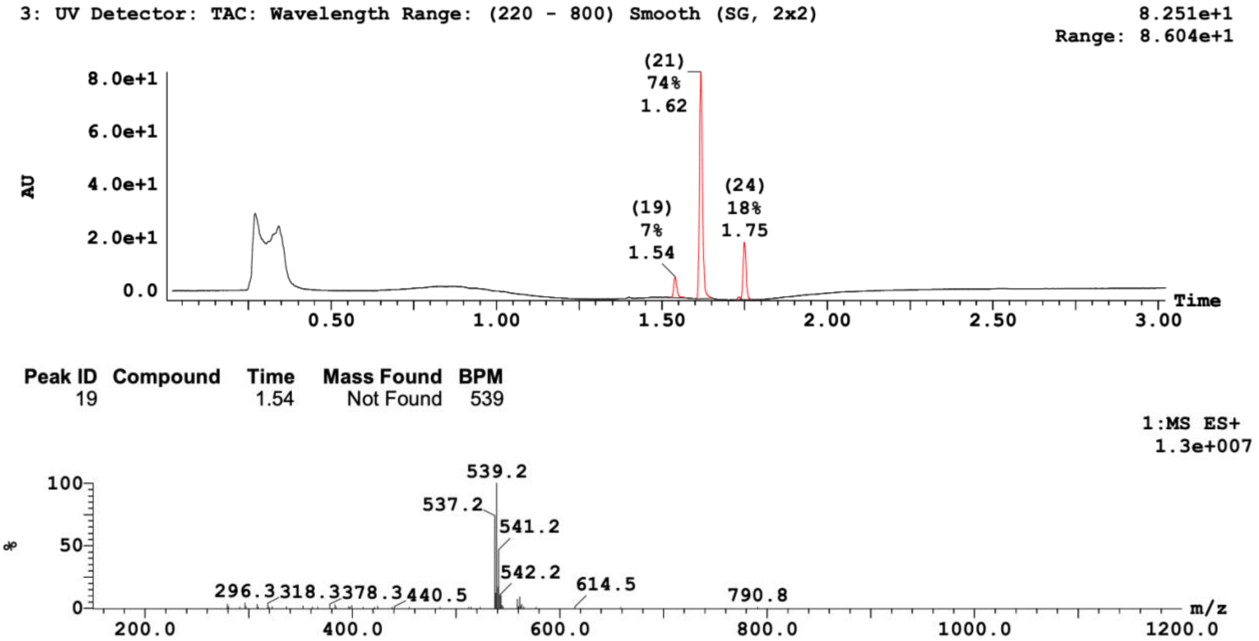

3 (1 eq) + *N*α-acetyl-*L*-lysine methyl ester hydrochloride (2 eq) + Tris (4 eq), 2h, rt, ^{19}F NMR

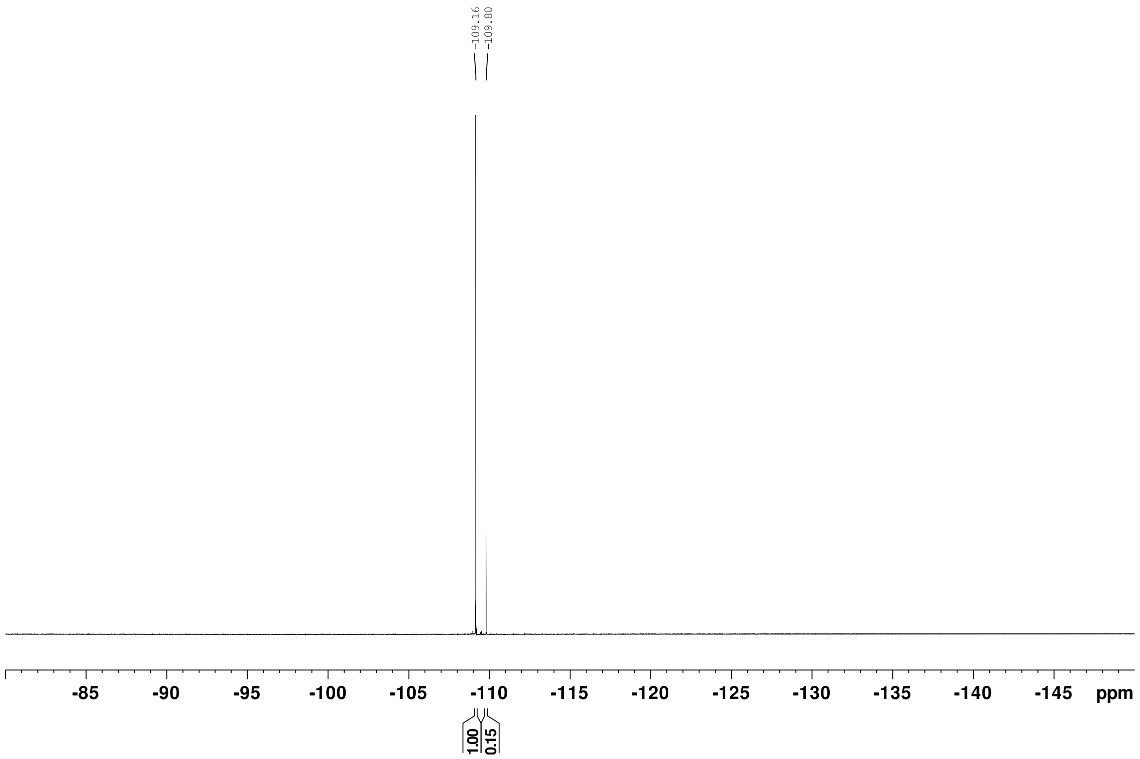

3 NMR Spectra

1, ^{13}C NMR (126 MHz, $CDCl_3$)

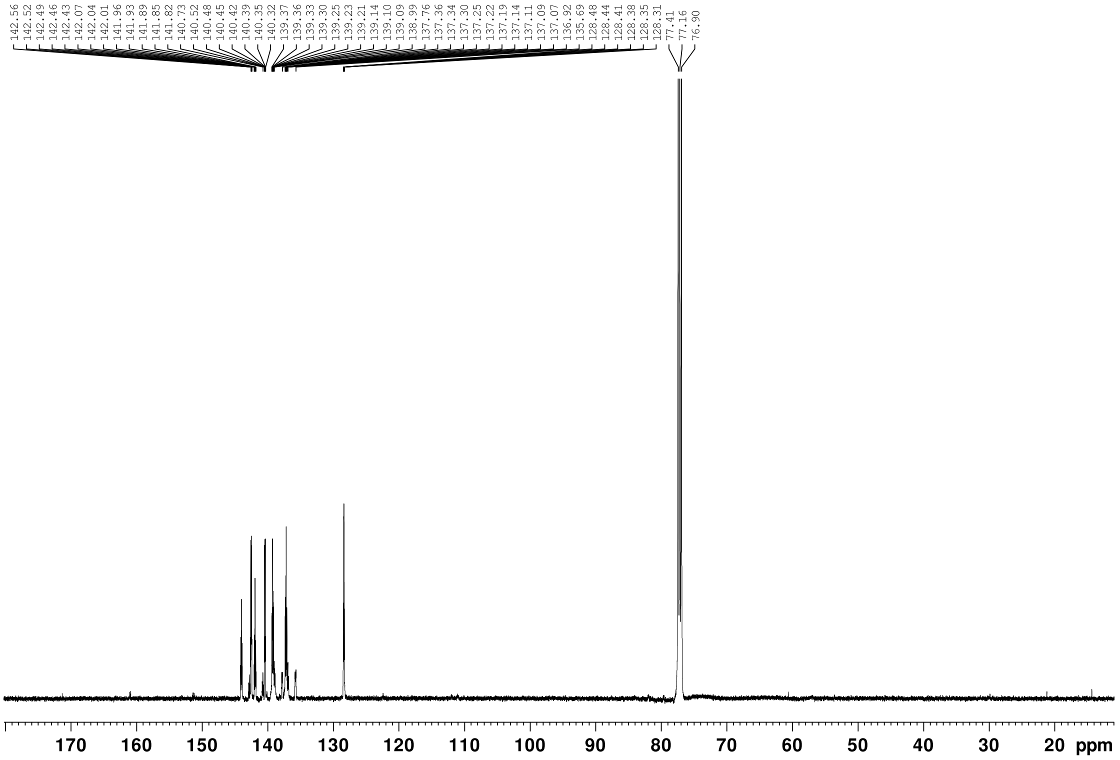

1, ^{19}F NMR (376 MHz, $CDCl_3$)

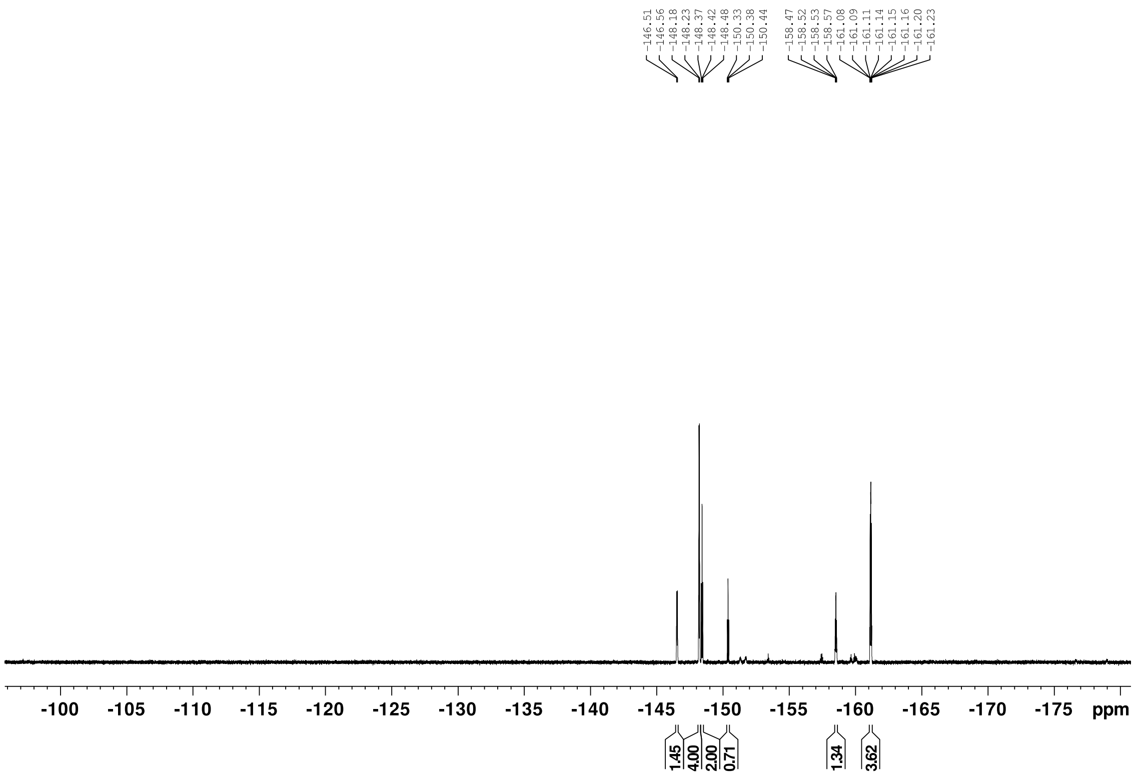

2, ^{1}H NMR (500 MHz, $CDCl_3$)

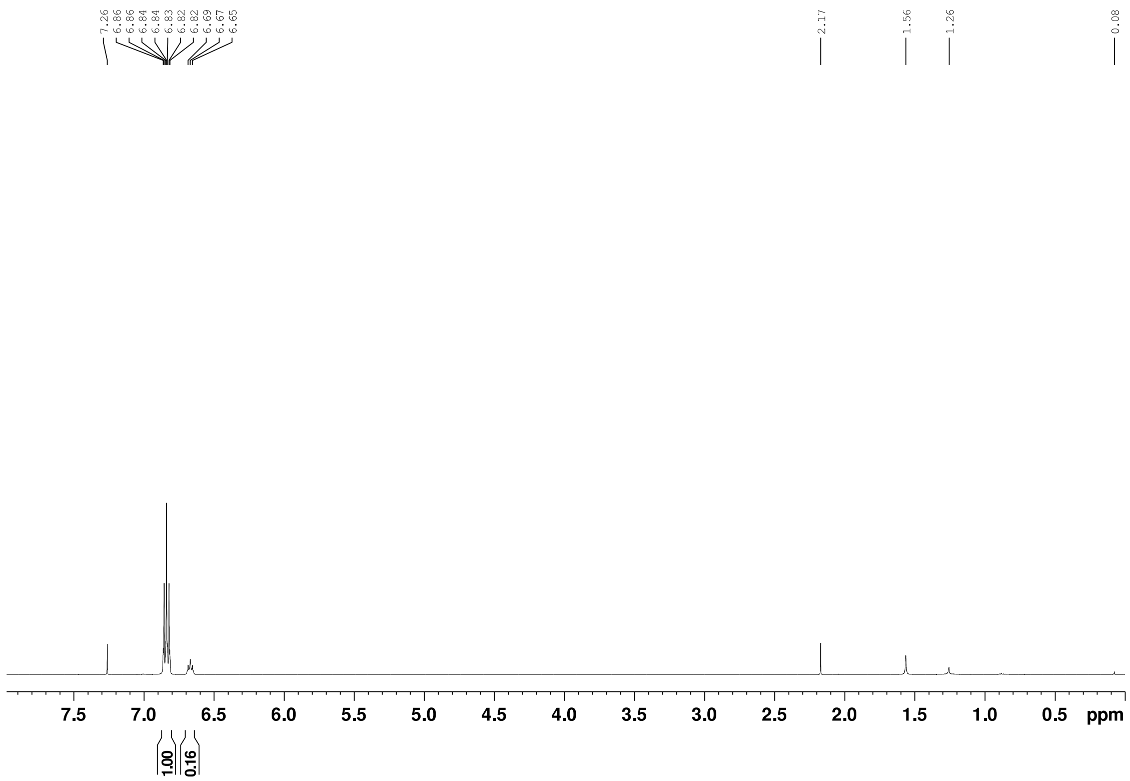

2, ^{13}C NMR (126 MHz, $CDCl_3$)

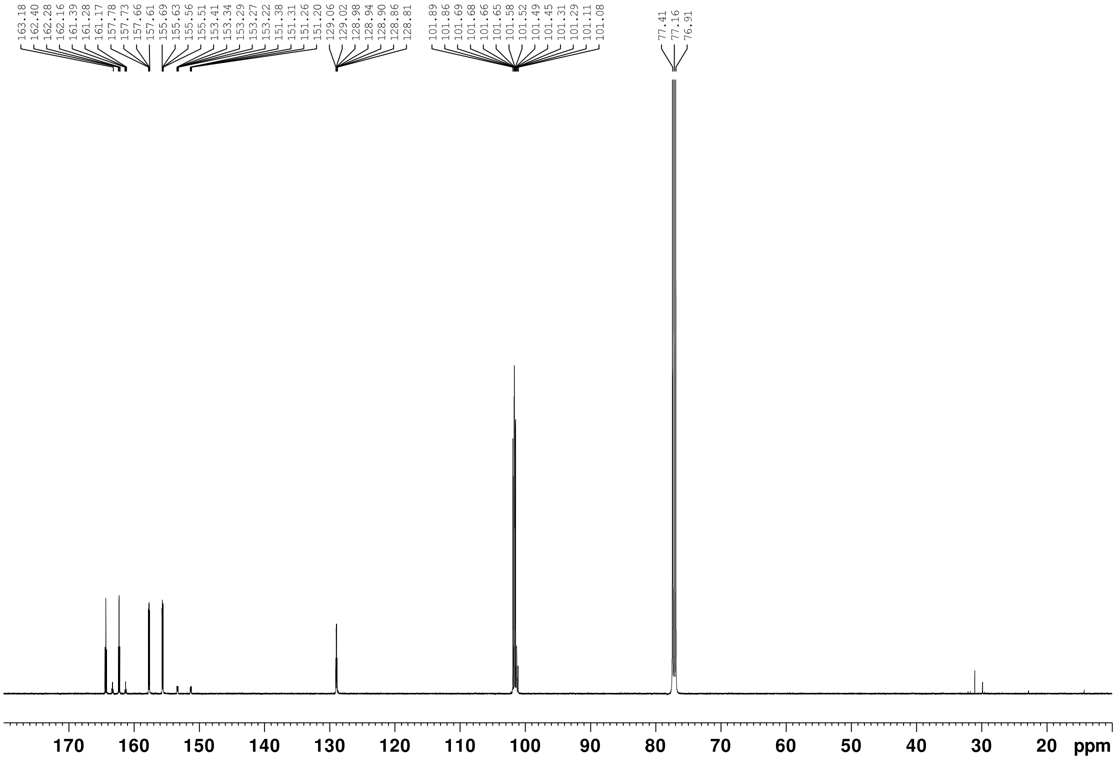

2, ^{19}F NMR (471 MHz, $CDCl_3$)

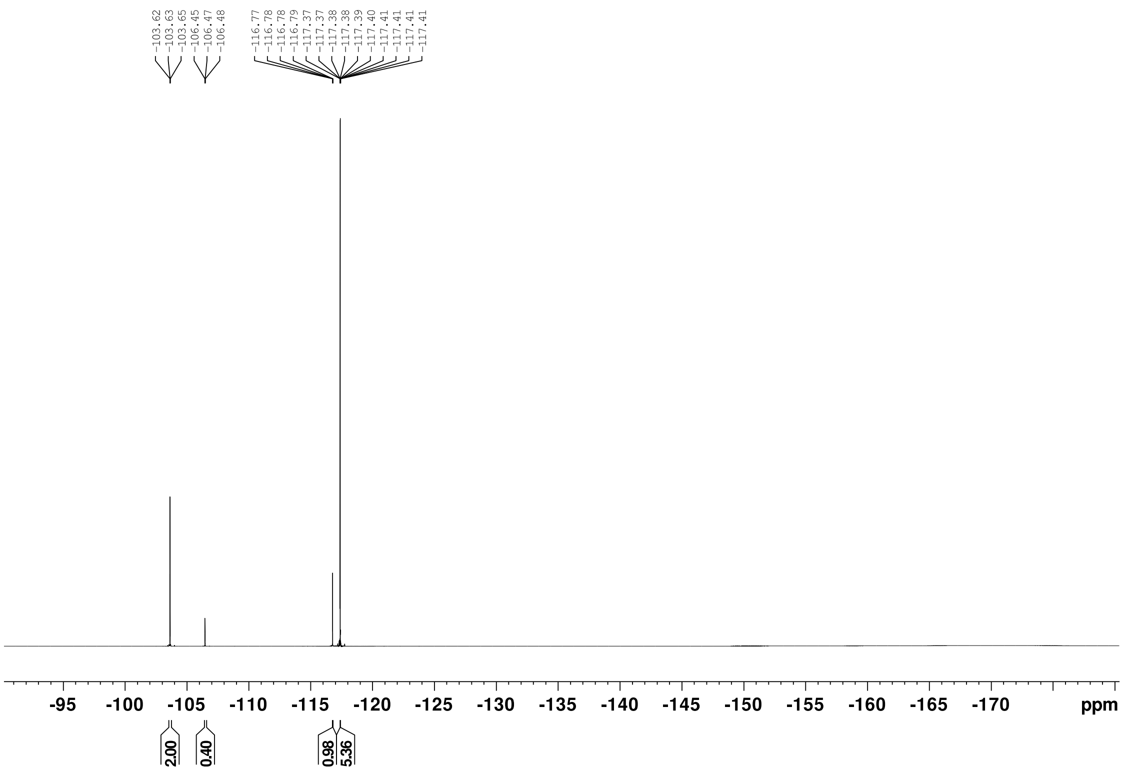

3, ^{1}H NMR (400 MHz, $CDCl_3$)

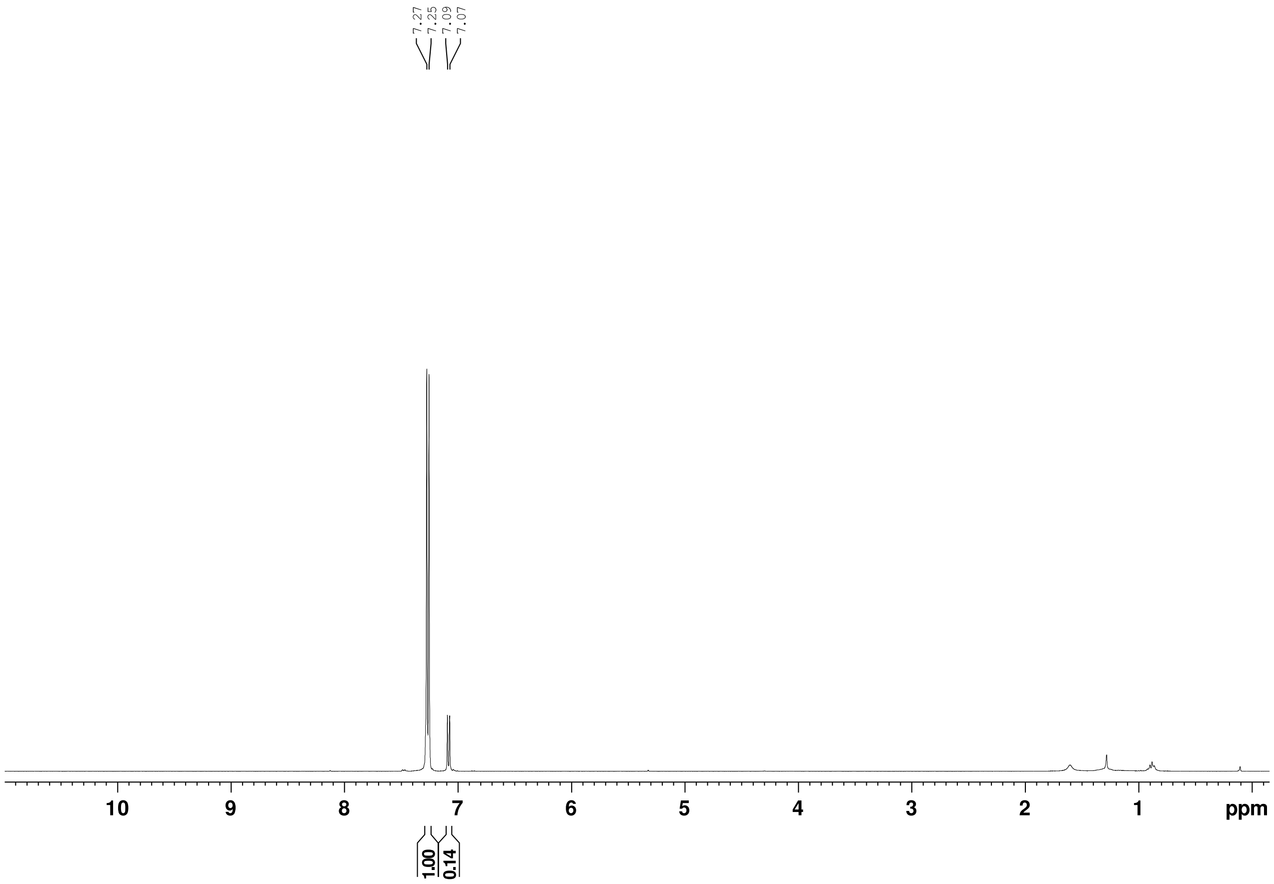

3, ^{13}C NMR (126 MHz, $CDCl_3$)

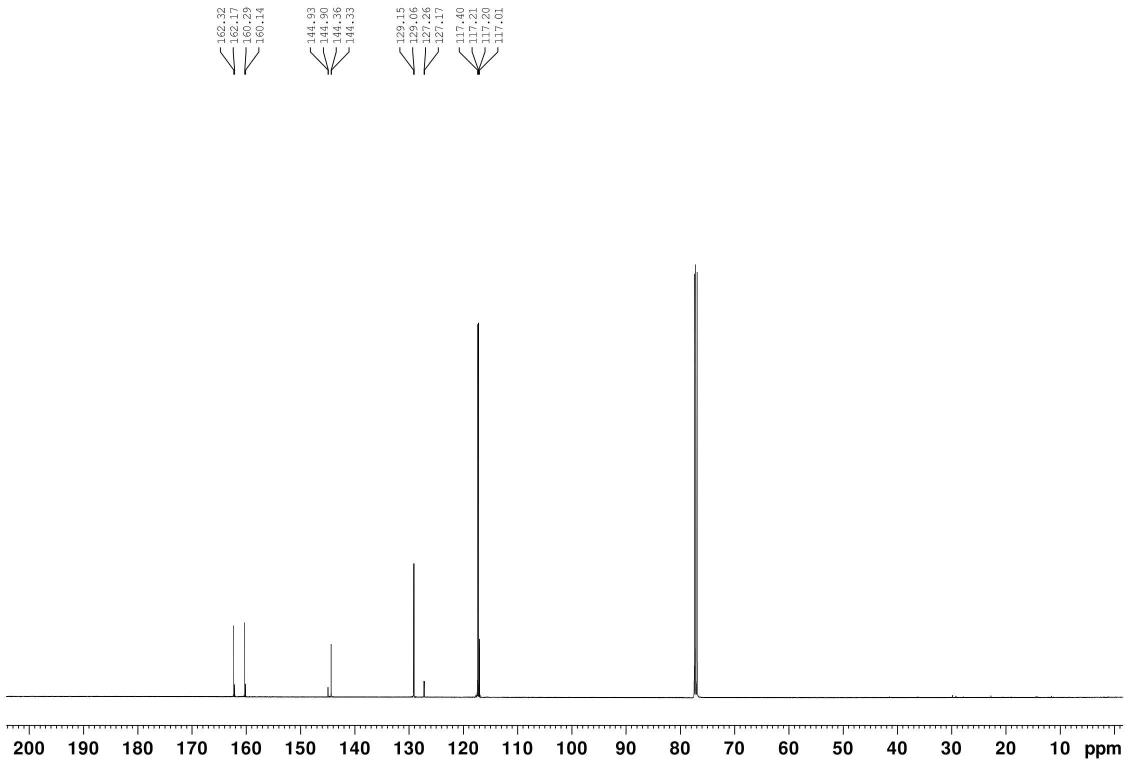

3, ^{19}F NMR (376 MHz, $CDCl_3$)

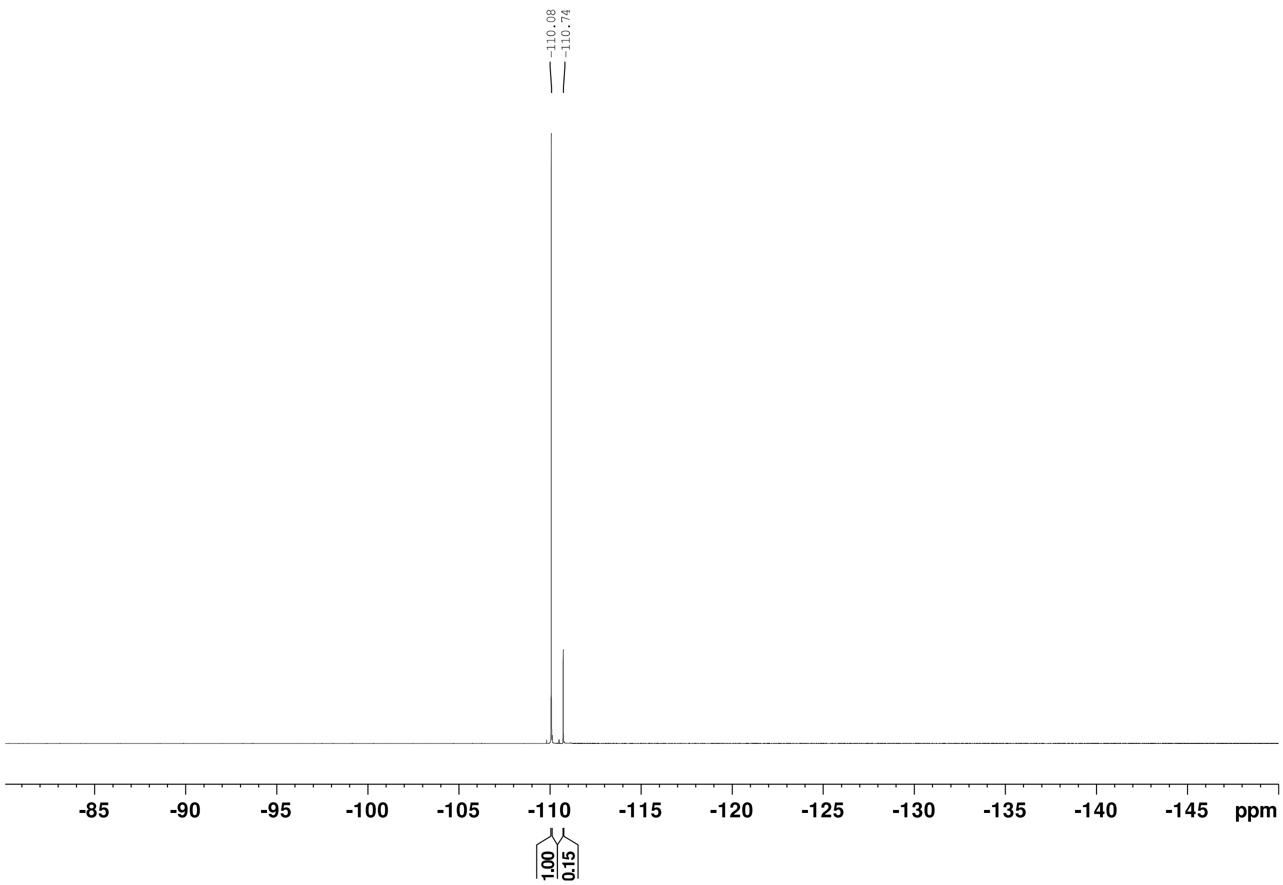

4, ^{1}H NMR (400 MHz, DMSO-d$_6$)

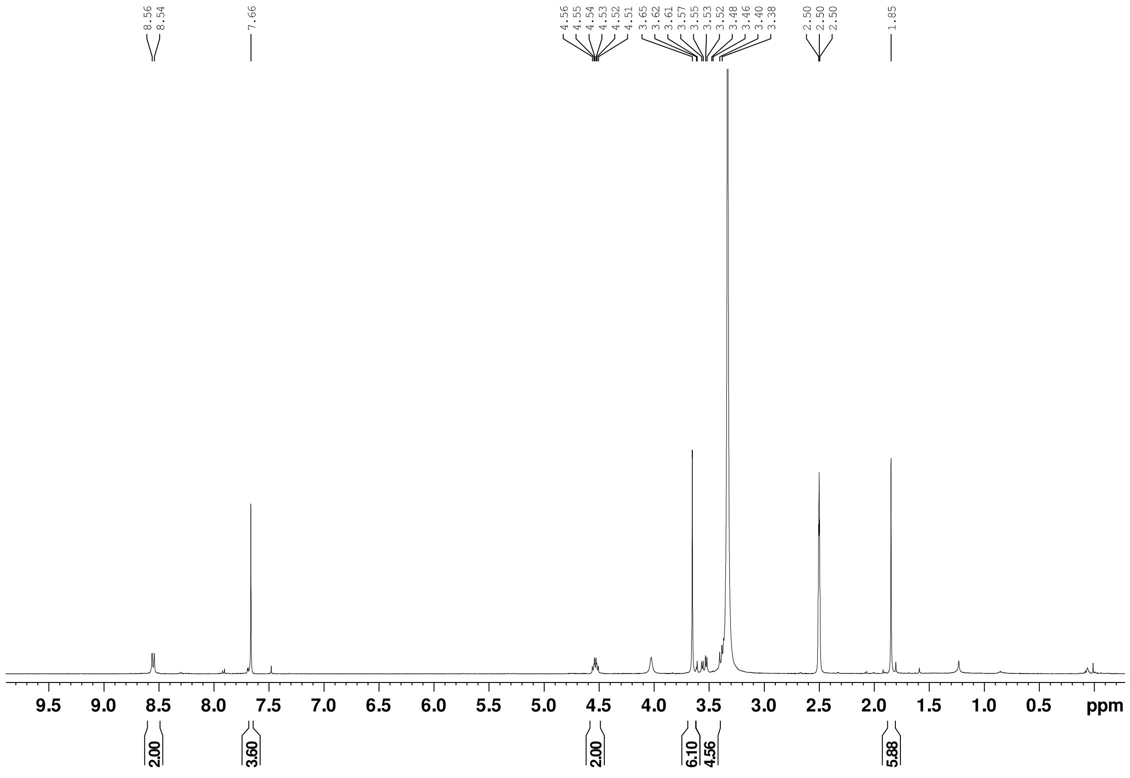

4, ^{13}C NMR (126 MHz, DMSO-d$_6$)

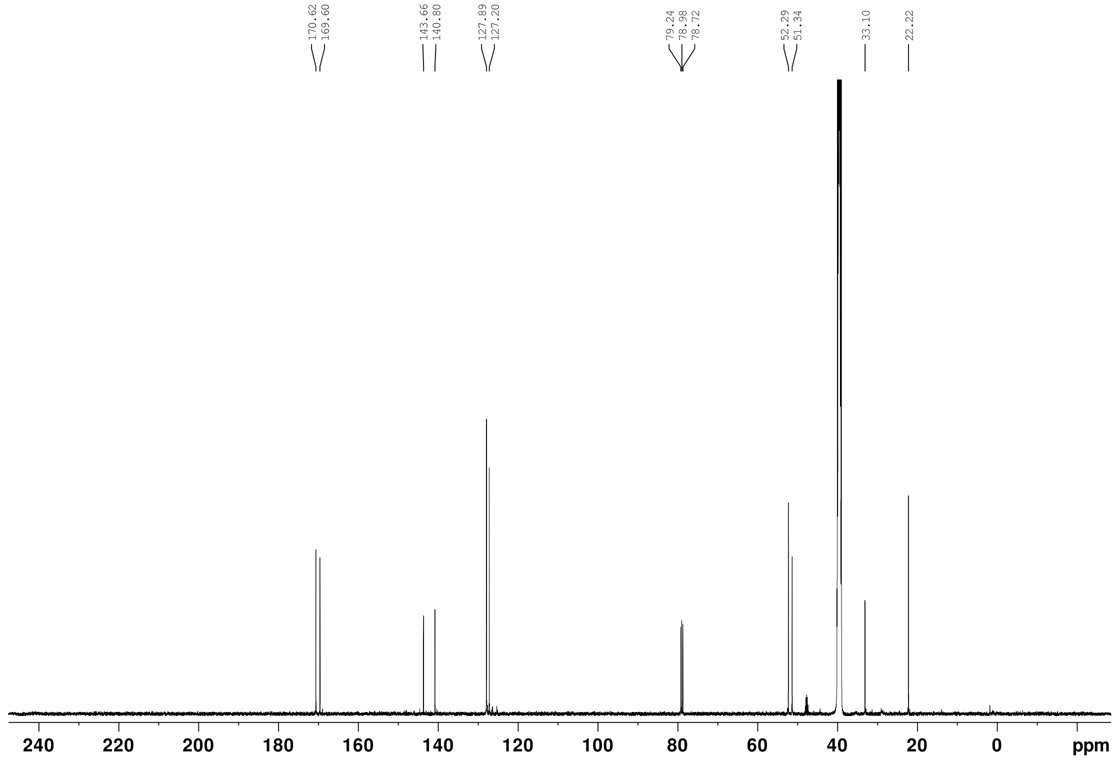

5a, ^{1}H NMR (400 MHz, $CDCl_3$)

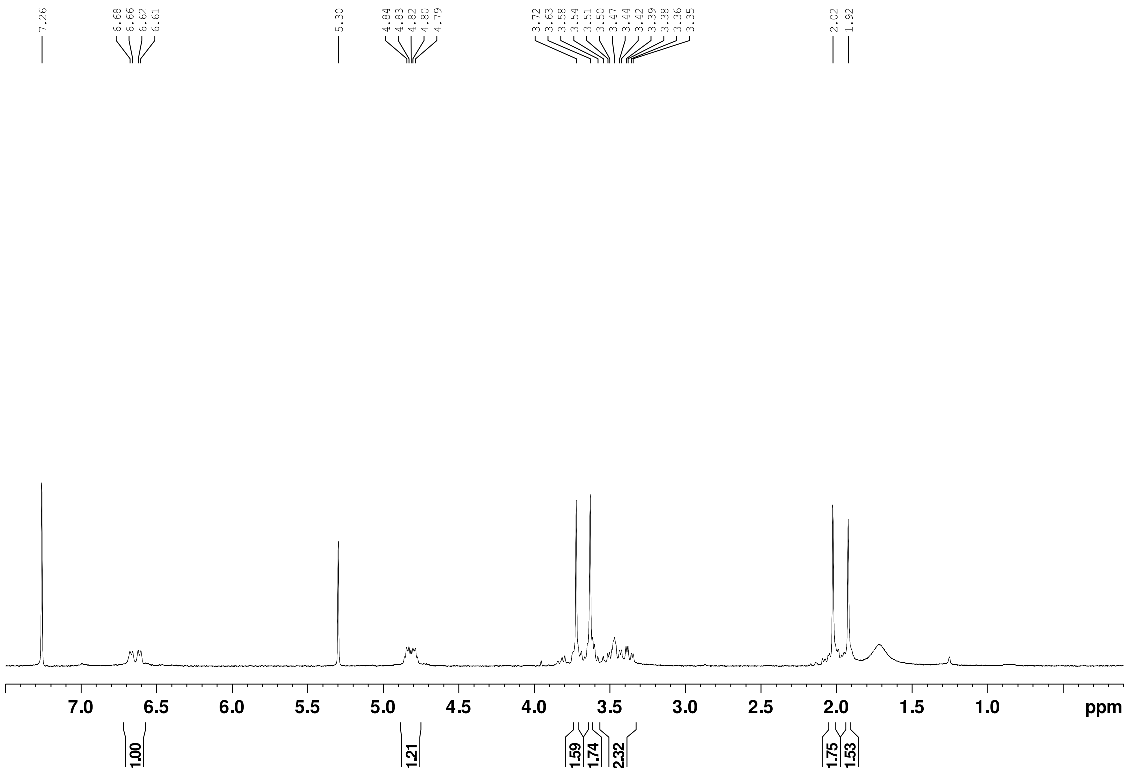

5a, ^{13}C NMR (101 MHz, $CDCl_3$)

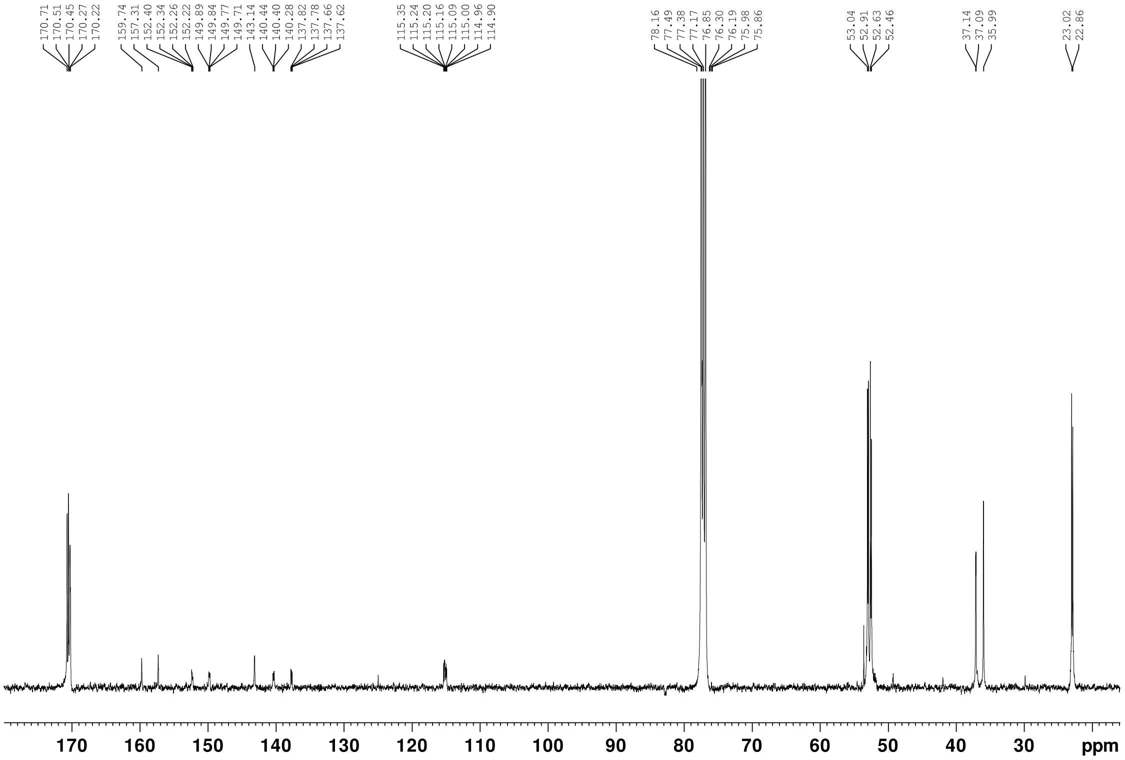

5a, ^{19}F NMR (376 MHz, $CDCl_3$)

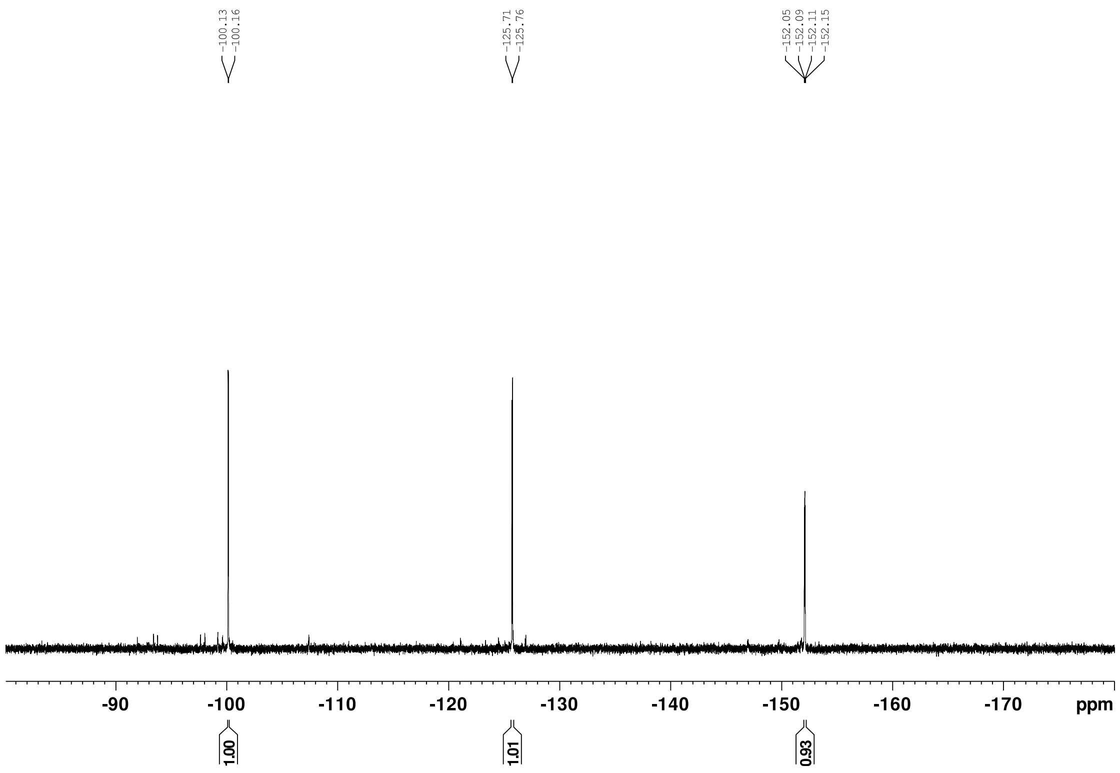

5b, ^{1}H NMR (500 MHz, $CDCl_3$)

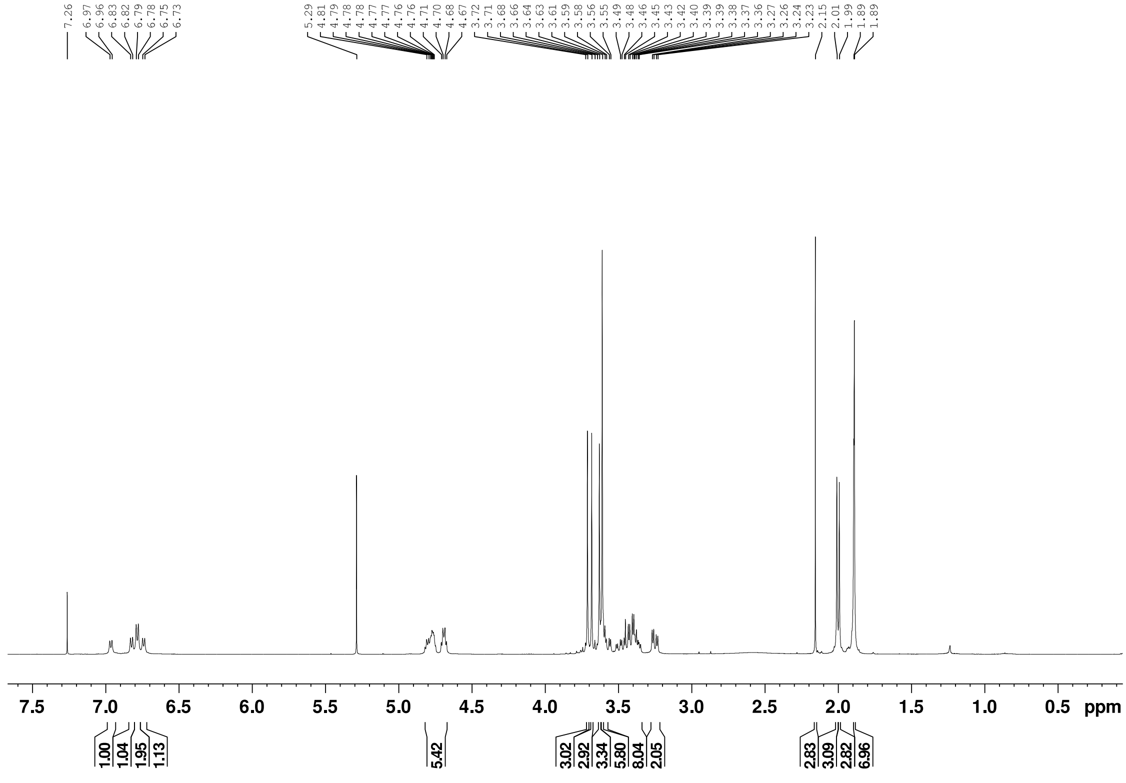

5b, ^{13}C NMR (126 MHz, $CDCl_3$)

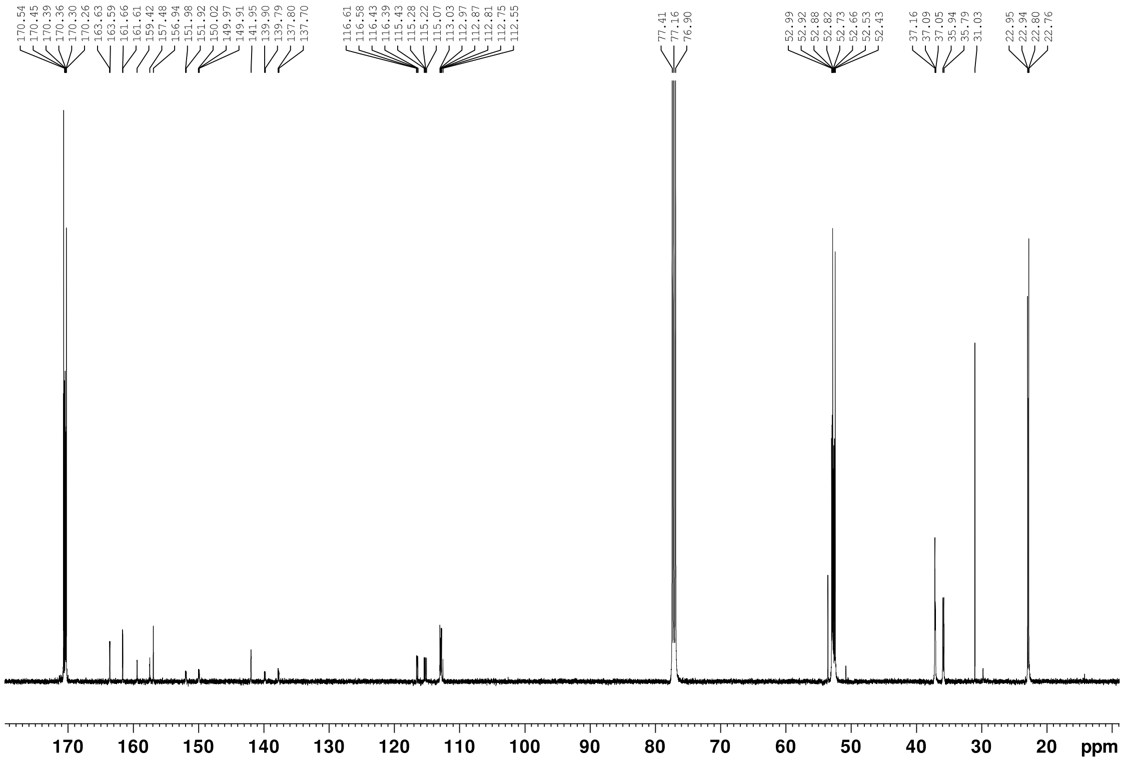

5b, ^{19}F NMR (376 MHz, $CDCl_3$)

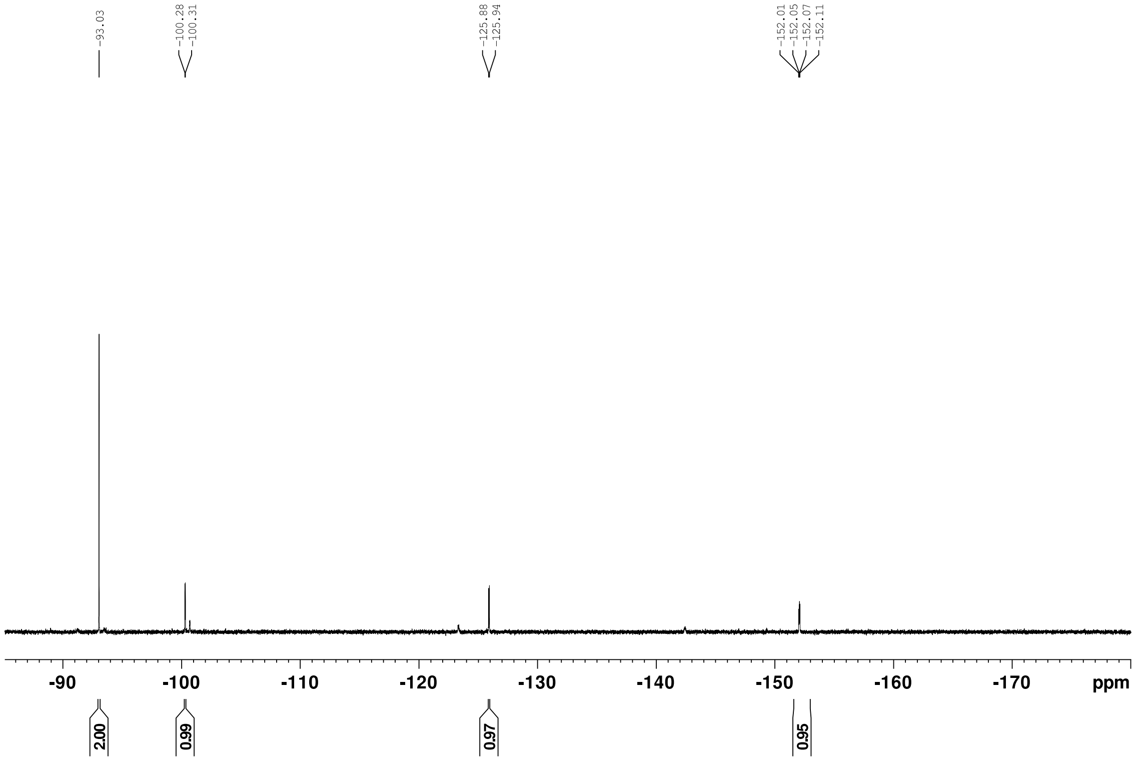

4 Peptide synthesis, analysis, and stapling procedures

4.1 Peptide Synthesis

General solid phase peptide synthesis (SPPS): Peptide synthesis was carried out on a 0.1 or 0.25 mmol scale using Rink Amide MBHA low-loading resin (0.308 mmol/g, 100-200 mesh) to afford *C*-terminal amides upon cleavage. An Fmoc protecting amino acid strategy was employed, with Trt (Asn, Cys), *t*-Bu (Asp, Glu, Ser, Thr, Tyr), Boc (Trp) and Pbf (Arg) side-chain protection.

Manual Fmoc-SPPS: Amino acid coupling was performed using Fmoc-protected amino acids (3 eq), HATU (3 eq) and DIPEA (6 eq) in DMF for 1-3 h. The completion of peptide coupling was determined using a Kaiser test, where colourless resin indicated complete coupling and blue-coloured resin indicated incomplete coupling. A second round of coupling was carried out for any incomplete coupling reactions. Fmoc deprotection was carried out using 20% piperidine in DMF (2 x 10 min).

Automated Fmoc-SPPS: Automated SPPS was carried out using CEM Liberty Blue® automated microwave peptide synthesiser. Amino acid coupling was performed using Fmoc-protected amino acids (5 eq), oxyma (10 eq) and DIC (5 eq) in DMF, using 25 W power at 75 °C, over 15 min. Fmoc deprotection was carried our using 20% piperidine in DMF using 45 W power at 75 °C, over 3 min.

Peptide capping: *N*-terminal capping of resin-bound peptide was achieved *via* acetylation using acetic anhydride (20 eq) and *N,N*-diisopropylethylamine (40 eq) in DCM at rt for 2 h. In a similar manner, capping of tracer peptide was achieved on resin, upon pre-activation of 5-carboxytetramethylrhodamine (5-TAMRA) (4 eq) with diisopropylcarbodiimide (4 eq) and HATU (8 eq), in DMF at rt overnight, to yield FP tracer peptide in a 15% yield.

Resin cleavage: Side chain deprotection and cleavage from resin for non-cysteine containing peptides was achieved using a TFA cleavage cocktail containing 2.5% triisopropylsilane, 2.5% dichloromethane and 2.5% water in TFA for 2 h. Side chain deprotection and cleavage from resin for cysteine containing peptides was achieved using a TFA cleavage cocktail containing 1.5% triisopropylsilane, 1.5% dichloromethane, 1.5% water, 1.5% 3,6-dioxa-1,8-octanedithiol (DODT) in TFA at rt for 3 h. Upon filtration and evaporation under a stream of N_2, the peptides were precipitated in ice-cold Et_2O. The crude peptides were purified by prep HPLC. The purified peptides were lyophilised, the purity determined by analytical HPLC and mass analysed by LCMS (**Table S5-S7**).

General procedure for peptide stapling: 3 (1 eq) was incubated with **P1** (1 mM), TRIS (tris(hydroxymethyl)aminomethane) base (10 eq) and TCEP•HCl (100 eq) at room temperature in DMF over

24-48 h to yield the stapled peptide **SP1** in 18-38% yield upon HPLC purification. DMF was degassed with N_2 for 10 minutes prior to being used in stapling.

4.2 Peptide Analysis

Table S5. Peptide sequence, analytical HPLC retention time and purity. Ac = acetyl capped, TAMRA = 5-carboxytetramethylrhodamine, X = stapled with linker **3**.

Peptide	Sequence	R_t (min)†	R_t (min)‡	% Purity (%)†
**PMI**	Ac-TSFAEYWNNLSP-NH_2	8.594	8.993	96
**FP tracer**	TAMRA-RFMDYWEGL-NH_2	8.845	9.338	95
**P1**	Ac-TSFACYWNGLSC-NH_2	7.531	8.008	91
**SP1**	Ac-TSFAXYWNGLSX-NH_2	9.753*<br>10.056**	10.453*<br>10.892**	99

†R_t on a 5-95% B gradient (A: 0.05% (v/v) TFA in H_2O, B: 0.05% (v/v) TFA in MeCN) over 18 min; ‡R_t on a 10-80% B gradient (A: 0.05% (v/v) TFA in H_2O, B: 0.05% (v/v) TFA in MeCN) over 18 min; **cis* isomer; ***trans* isomer.

Table S6. Mass of peptides determined by LCMS analysis.

Peptide	Exact mass	m/z found	m/z calc	Species
**PMI**	1467.70	735.1	734.9	$[M+2H]^{2+}$
**FP tracer**	1626.70	814.6	814.4	$[M+2H]^{2+}$
**P1**	1391.56	697.0	696.8	$[M+2H]^{2+}$
**SP1**	1705.46	852.8	851.7	$[M-2H]^{2-}$

Table S7. SPPS yields of linear peptides upon HPLC purification.

Peptide	Amount of resin used (mg)	Amount of resin used (mmol)	Yield of peptide (mg)	Yield of peptide (%)
**PMI**	812	0.25	23	6
**P1**	812	0.25	49	14

4.3 LCMS Spectra

PMI

Peak ID	Compound	Time	Mass Found	BPM
9		1.24	Not Found	735

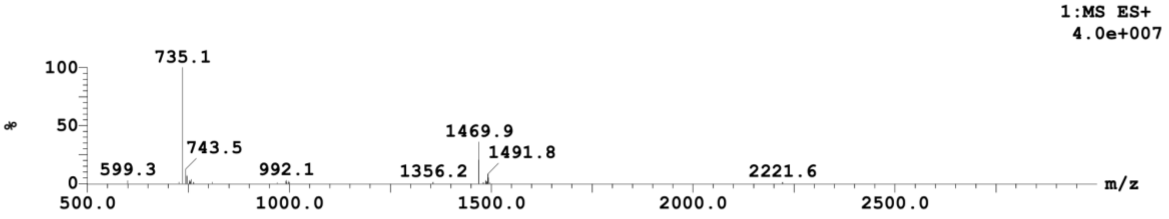

FP tracer

Peak ID	Compound	Time	Mass Found	BPM
4		1.15	Not Found	815

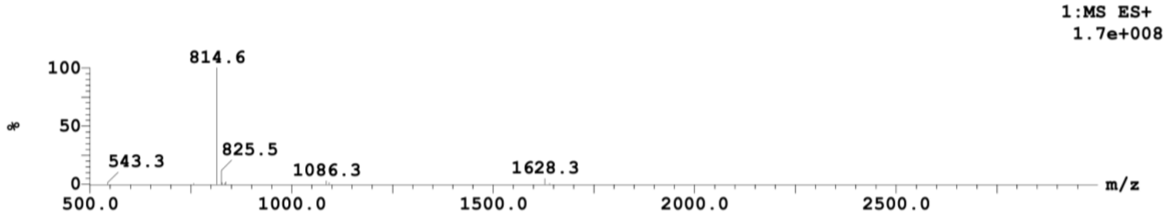

P1

Peak ID	Compound	Time	Mass Found	BPM
5		1.12	Not Found	697

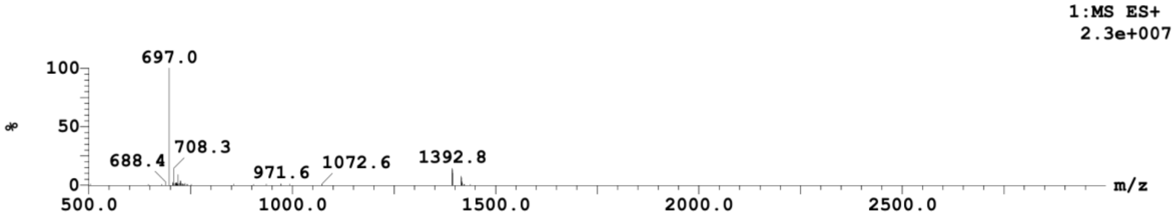

SP1

Peak ID	Compound	Time	Mass Found	BPM
7		1.33	Not Found	853

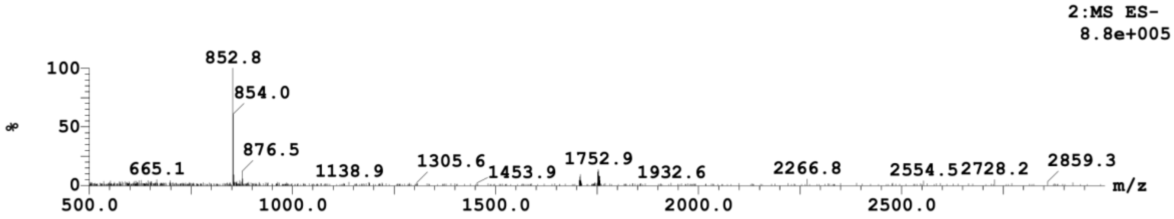

4.4 HPLC Spectra

PMI 5-95% B (220 nm and 254 nm)

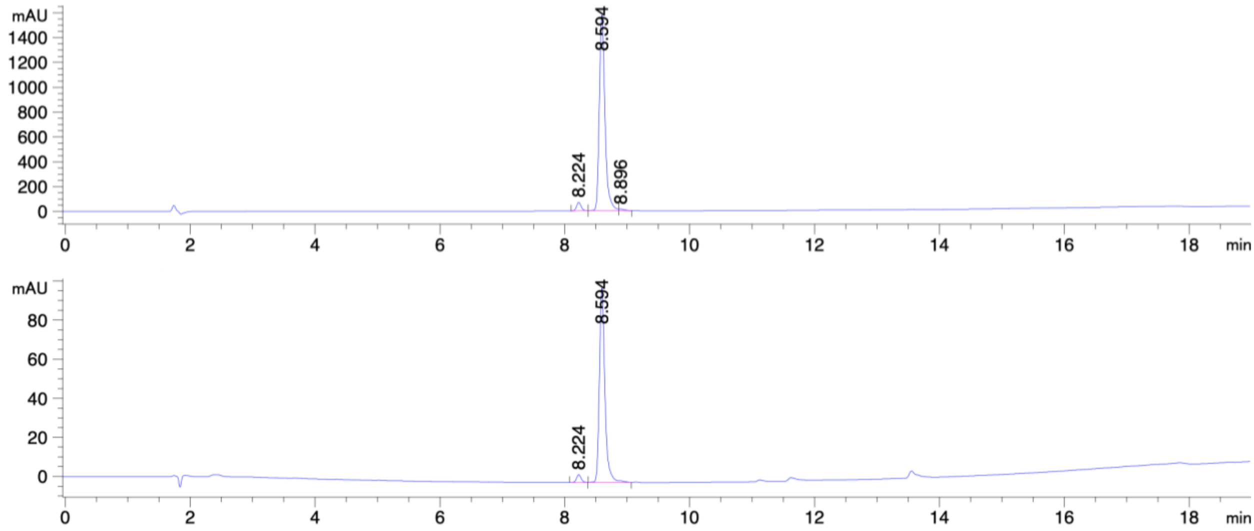

PMI 10-80% B (220 nm and 254 nm)

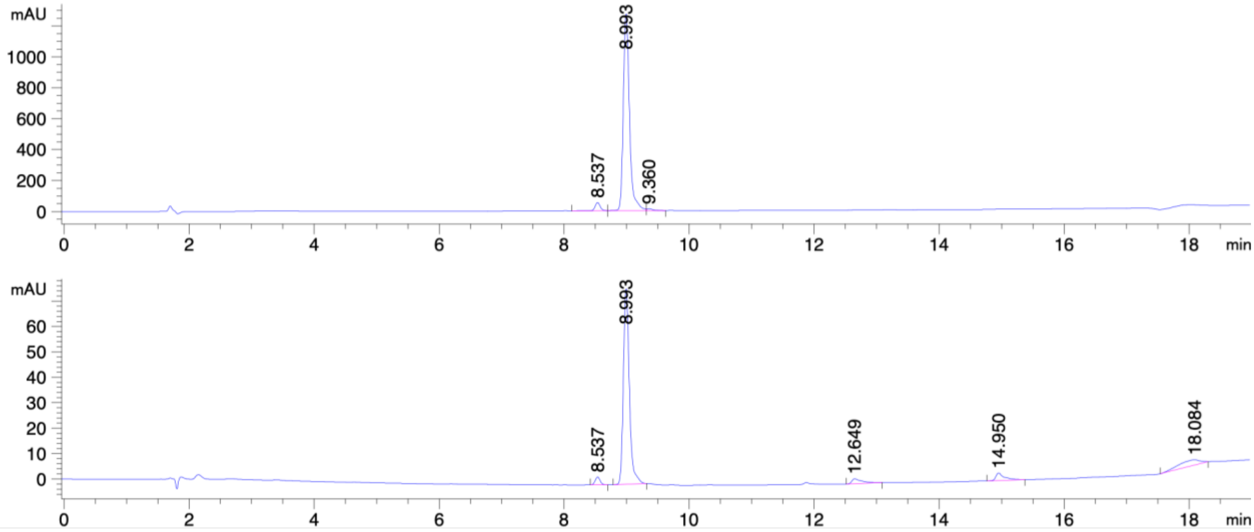

FP tracer 5-95% B (220 nm and 254 nm)

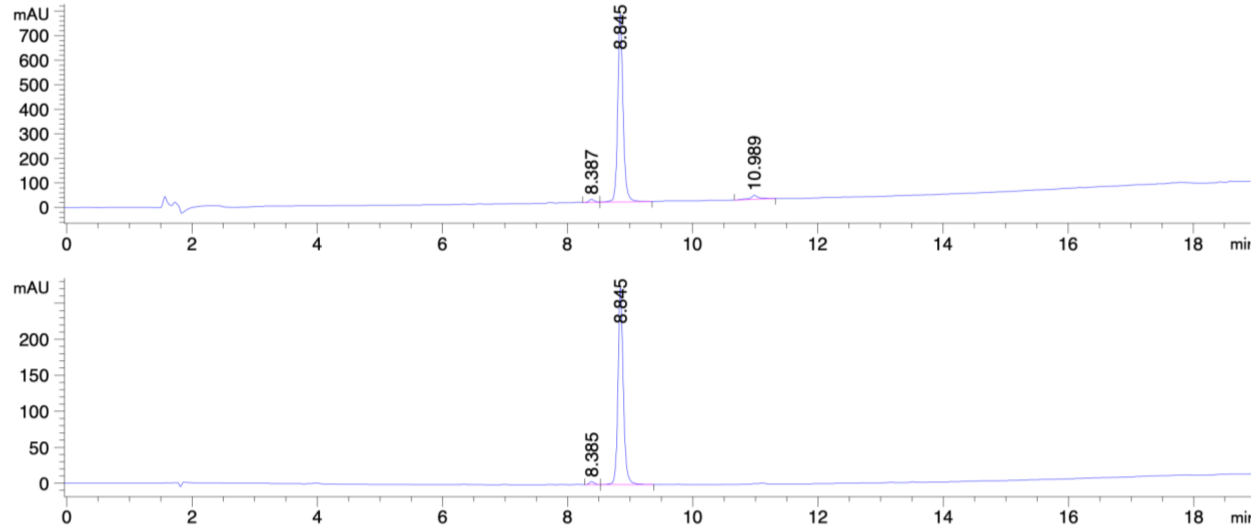

FP tracer 10-80% B (220 nm and 254 nm)

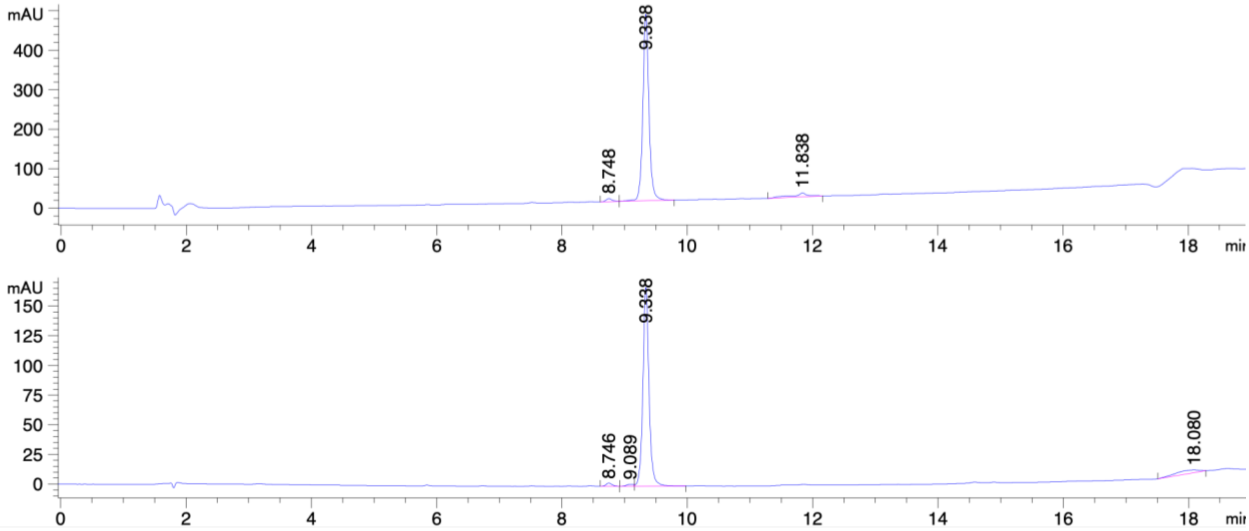

P1 5-95% B (220 nm and 254 nm)

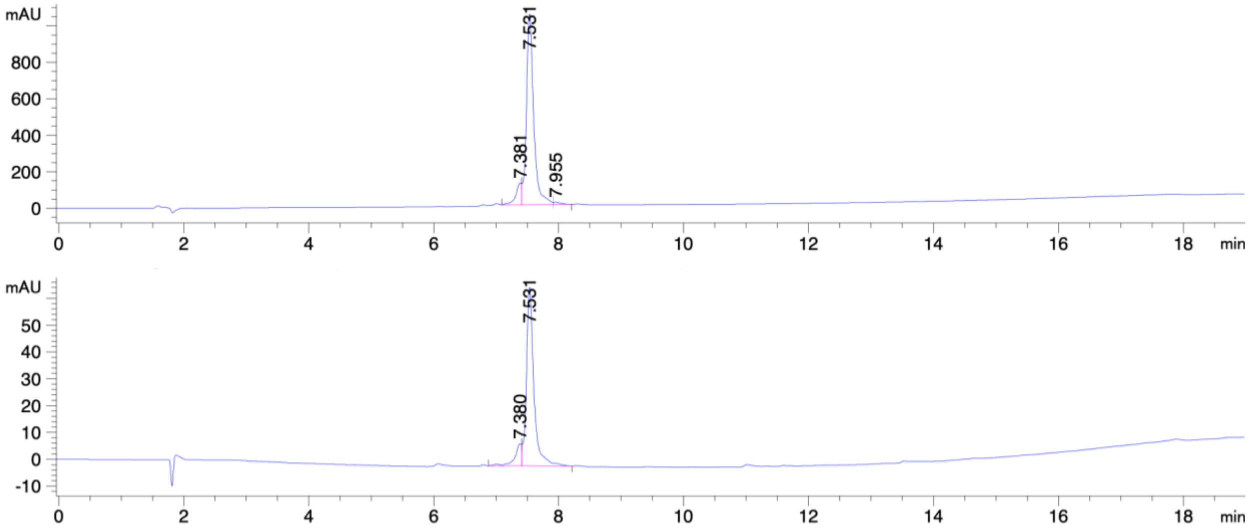

P1 10-80% B (220 nm and 254 nm)

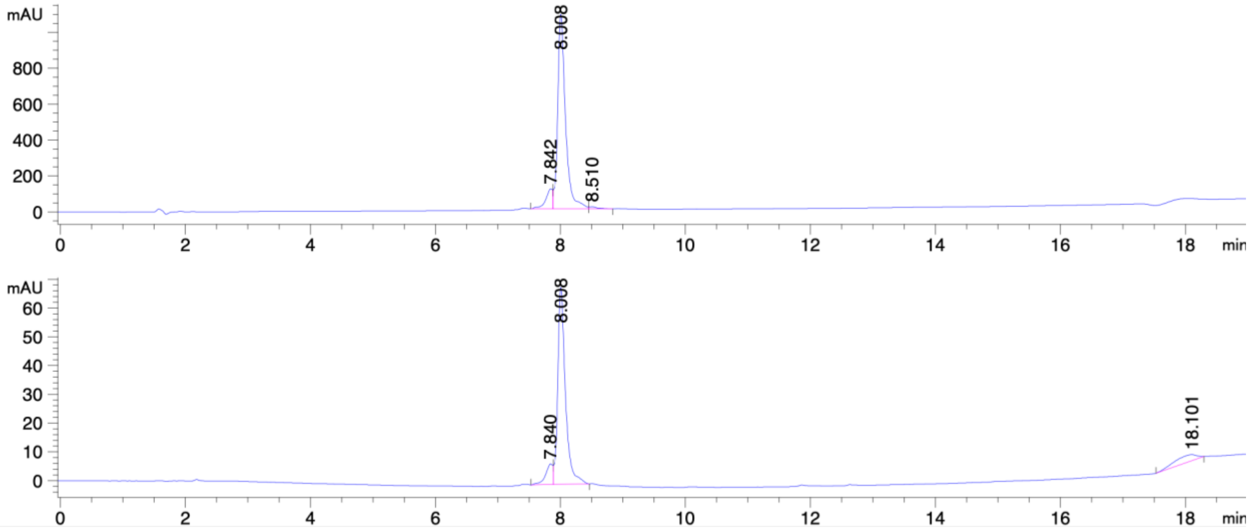

SP1 5-95% B (220 nm and 280 nm) in DMSO

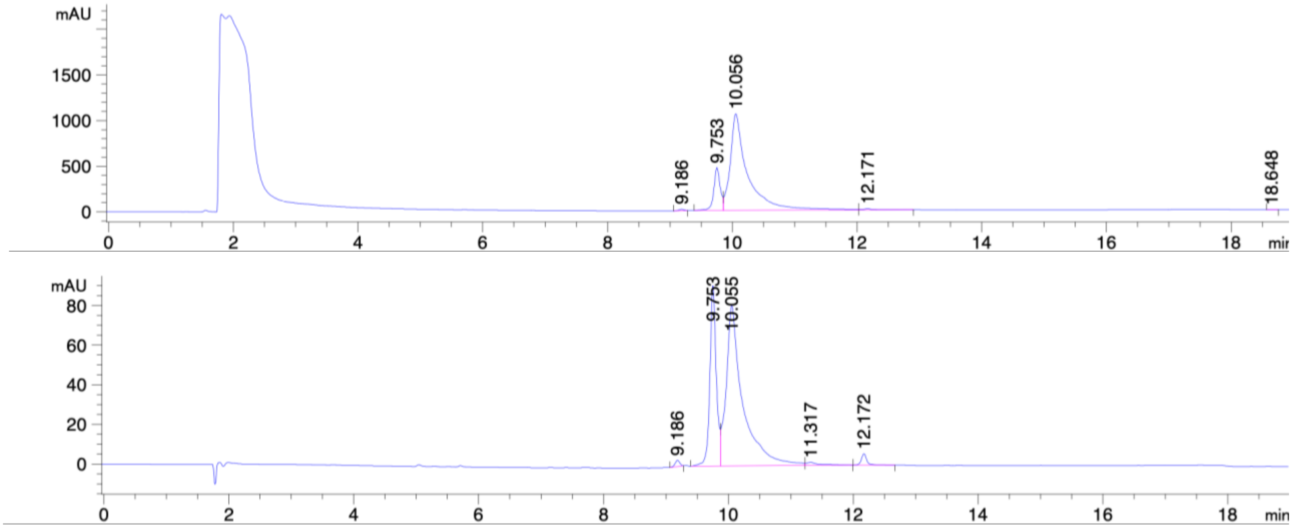

SP1 10-80% B (220 nm and 280 nm) in DMSO

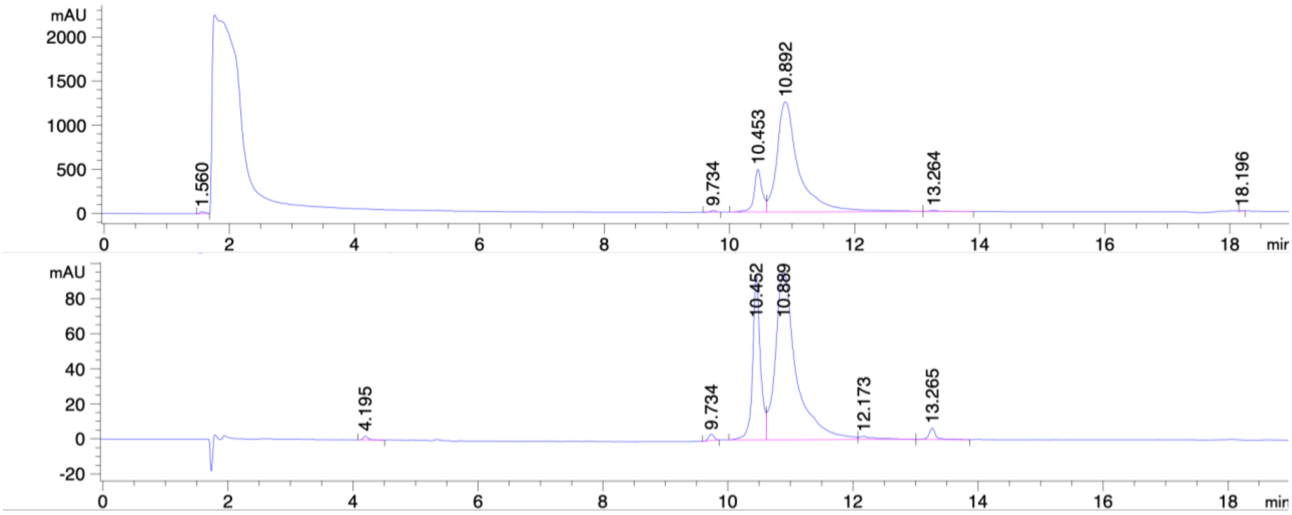

5 Characterisation of Isomerisation

5.1 Light-Induced Isomerisation Studies

Photoisomerisation studies were carried out using Thorlabs Single-Color Visible Mounted LEDs of 415 and 660 nm wavelengths, with an irradiance of 15.6 and 20.88 μW/mm^2, respectively (Figure S1 and S2).

6 Stability Studies

6.1 Half-Life Studies

The half-life of the metastable *cis* **SP1** form in FP assay buffer was determined by analytical HPLC analysis (Figure S3). The measurements were carried out in two independent experiments.

6.2 Serum Stability Studies

P1 and **SP1** (10 μL, 10 mM stock in DMSO) were incubated with PBS (1x, 500 μL), human serum (50 μL) and caffeine (10 μL of a 11.25 mg/mL solution in MQ water) at 37 °C for 5 days. At specific intervals, aliquots (25 μL) of the reaction mixture were taken, flash frozen in liquid nitrogen and stored at -20 °C prior to analysis on the same day. Upon unfreezing, the samples were quenched with 25 μL of 96% ethanol and 25 μL of DMSO. The samples were spun at 13400 g for 10 minutes and the supernatant analysed by HPLC analysis, with a 5-95% B gradient (A: 0.05% (v/v) TFA in H_2O, B: 0.05% (v/v) TFA in MeCN) over 18 minutes at 220 nm. Caffeine was used as an internal standard to enable quantification of degradation over time. The experiment was performed in duplicates (Table S2-4).

7 Fluorescence Polarisation (FP) Assay

The assays were carried out in accordance with previously reported literature, using purified MDM2 protein and 5-TAMRA labelled FP tracer.[2,3] The fluorescence polarisation measurements were carried out after 20 minutes of incubation using a Clariostar (BMG) plate reader using a excitation filter (540 nm) and emission filter at 590 nm both with a 20 nm bandwidth and a 566 nm dichroic filter. Assay buffer (PBS, 0.05% (v/v) Tween-20, 3% (v/v) DMSO) was used as a negative control in place of peptide, while assay buffer in place of MDM2 and peptide was used as the positive control. The measurements were carried out in two or three independent experiments, each as a triplicate. All the values are reported with the standard error of the mean.

7.1 MDM2 Fluorescence Polarisation Binding Assay

A stock solution of MDM2 (195 μM) was diluted in assay buffer (PBS, 0.05% (v/v) Tween-20, 3% (v/v) DMSO) to 19.5 μM (9.75 μM highest concentration) and was diluted across the 384-well plate to give an 18-point

dose-response curve. TAMRA-labelled peptide (TAMRA-RFMDYWEGL-NH_2; 10 mM) in DMSO was diluted in assay buffer to 30 nM and 10 μl added to each well (15 nM final assay concentration). The fluorescence polarisation measurements were subsequently carried out.

The *K*d value of FP tracer peptide was calculated to be 7.6 nM using a using a 1:1 binding model in the GraphPad Prism software, using the equations previously described by Brown *et al.* for competitive binding where both the target protein and the peptide binding are depleted in a stoichiometric manner over time during the period of incubation (Figure S4). In the equations used, r = anisotropy measured, r_0 = anisotropy of free peptide, r_b = anisotropy of MDM2:5-TAMRA peptide complex, K_{d1} = dissociation constant of 5-TAMRA peptide to MDM2, K_{d2} = (apparent) dissociation constant of non-labelled ligand to MDM2, $[P]_t$ = MDM2 concentration, $[L]_t$ = non-labelled ligand concentration and $[L]_{st}$ = 5-TAMRA peptide concentration.

$$r = r_0 + (r_b + r_0) \times \frac{2\sqrt{(d^2 - 3e)}\cos\left(\frac{\theta}{3}\right) - 9}{3K_{d1} + 2\sqrt{(d^2 - 3e)}\cos\left(\frac{\theta}{3}\right) - d}$$

$$d = K_{d1} + K_{d2} + [L]_{st} + [L]_t - [P]_t$$

$$e = K_{d1}([L]_t - [P]_t) + K_{d2}([L]_{st} - [P]_t) + K_{d1}K_{d2}$$

$$\theta = cos^{-1}\left(\frac{-2d^3 + 9de - 27f}{2\sqrt{(d^2 - 3e)^3}}\right)$$

$$f = -K_{d1}K_{d2}[P]_t$$

7.2 MDM2 Competitive Fluorescence Polarisation Binding Assay

Peptide stock solutions (10 mM) were prepared in DMSO and diluted in assay buffer (PBS, 0.05% (v/v) Tween-20, 3% (v/v) DMSO) to a top concentration of 200 µM for PMI derivative and 250 µM for **P1** and **SP1**, giving a final assay concentration of 100 or 125 µM, respectively). Subsequently, 2-fold serial dilutions were carried out across the 384-well plate to give a 23-point dose-response curve. To each well containing 10 µL of peptide sample in assay buffer, 10 µL of a mixture of MDM2 and FP tracer in buffer were added, to give a final concentration of 97.5 nM MDM2 and 50 nM of FP tracer peptide. The fluorescence polarisation measurements were subsequently carried out (Figure S5). GraphPad Prism was used to determine the K_i using the K_d of the FP tracer peptide determined in section 6.1. Peptides **PMI** and **P1** were fitted to nonlinear regression (curve fit) for competitive one site receptor binding and **SP1** was fitted to nonlinear regression (curve fit) for competitive two site receptor binding. *In situ* isomerisation of **SP1** was enabled by red light (λ

= 660 nm, 20.88 μW/mm^2, 90 minutes) or purple light (λ = 415 nm, 15.60 μW/mm^2, 30 minutes) irradiation of peptide in FP buffer prior to addition of MDM2 and FP tracer peptide.

8 Circular Dichroism (CD) Spectroscopy

Approximately, 0.1 mg/mL peptide in 1:1 H_2O/MeCN or MeCN was used to obtain circular dichroism spectra over a 190-250 nm range at 25 °C. *In situ* isomerisation of **SP1** was achieved by irradiating the sample with red light (λ = 660 nm, 20.88 μW/mm^2, 90 minutes) and purple light (λ = 415 nm, 15.60 μW/mm^2, 30 minutes). The CD spectra were acquired using an AVIV 410 spectrometer at 25 °C, in 1 mm path-length cuvettes and in 1 nm wavelength steps averaged over 5 s/nm and a total of three scans which were baseline-corrected for solvent smoothing, using the manufacturer's software.

9 References

1 A. Antoine John and Q. Lin, *J. Org. Chem.*, 2017, **82**, 9873–9876.

2 C. J. Brown, S. T. Quah, J. Jong, A. M. Goh, P. C. Chiam, K. H. Khoo, M. L. Choong, M. A. Lee, L. Yurlova, K. Zolghadr, T. L. Joseph, C. S. Verma and D. P. Lane, *ACS Chem. Biol.*, 2013, **8**, 506–512.

3 Y. H. Lau, P. De Andrade, S. T. Quah, M. Rossmann, L. Laraia, N. Sköld, T. J. Sum, P. J. E. Rowling, T. L. Joseph, C. Verma, M. Hyvönen, L. S. Itzhaki, A. R. Venkitaraman, C. J. Brown, D. P. Lane and D. R. Spring, *Chem. Sci.*, 2014, **5**, 1804–1809.
